# Nanostructured Dual-Delivery System with Antioxidant and Synergistic Approach for Targeted Dermal Treatment

**DOI:** 10.3390/ijms26199485

**Published:** 2025-09-28

**Authors:** Lucia Dzurická, Julie Hoová, Barbora Dribňáková, Petra Skoumalová, Paola Rappelli, Ivana Márová

**Affiliations:** 1 Department of Food Science and Biotechnology, Faculty of Chemistry, Brno University of Technology, Purkyňova 464/118, 612 00 Brno, Czech Republic; 2 Department of Biomedical Sciences, Università Degli Studi di Sassari, Viale S. Pietro 43/B, 07100 Sassari, Italy

**Keywords:** synergism, phytochemicals, antimicrobial and antioxidant agents, wound healing, polyhydroxybutyrate, liposomes, nanofibres

## Abstract

Biocompatible nanofibrous dressings integrating bioactive compounds with antioxidative and antimicrobial properties offer a promising solution for effective wound healing. In the presented study, we developed a novel dual-delivery system by combining forcespun nanofibres with poly(3-hydroxybutyrate) (PHB)-liposomes to enhance bioavailability and enable targeted release of bioactive agents (eugenol, thymol, curcumin, ampicillin, streptomycin, gentamicin). These agents exhibited notable antioxidant activity (2.27–2.33 mmol TE/g) and synergistic or partially synergistic antimicrobial effects against *E. coli*, *M. luteus*, *S. epidermidis*, and *P. aeruginosa* ( Fractional Inhibitory Concentration index 0.09–0.73). The most potent combinations, particularly thymol, eugenol, and ampicillin, were encapsulated in the nanofibre–liposomal matrix. The successful preparation of a new combined delivery system was confirmed by structural analysis using Electron and Fluorescence Microscopy. The dual-composite materials retained the antimicrobial properties of the individual compounds upon release, with the highest increases of ~73.56% against *S. epidermidis*. Cell viability and in vitro immunology assays using the human keratinocyte cell line (HaCaT) showed a slight decrease in viability and immune response stimulation, while not impairing wound re-epithelisation. These findings highlight the potential of firstly reported novel carrier utilising both PHB-nanofibres and PHB-liposomes, exhibiting simultaneous antioxidant and antimicrobial activity as promising candidates for the treatment of infected wounds under oxidative stress.

## 1. Introduction

Wound healing is a dynamic and complex physiological process involving the regeneration and repair of damaged tissue that requires a suitable environment to facilitate the recovery. This process encompasses a series of organised, interdependent, and overlapping phases that begin immediately after the injury [1,2,3].

Microbial colonisation is an inherent feature of wounds. Wound infection is typically established when the microorganisms proliferate to levels when microorganisms proliferate to the point where their virulence factors overcome the host’s immune defences. The spread of infection can lead to tissue damage and delayed healing [3,4]. The composition of the wound microbiome is polymicrobial, consisting of both bacterial and fungal colonisation, which are believed to impede healing processes and contribute to the development of chronicity [5].

In order to avoid infections, a key approach is the use of topical antibiotics and antiseptics, as this strategy reduces side effects and occurrence of antimicrobial resistance that could be induced by drugs administered systemically. A dressing which provides direct release of antimicrobial agents at the wound site can effectively control microbial infection and furthermore protect the wound from secondary contamination. However, following the increasing occurrences of bacterial resistance, local antibiotics are being used with caution [3,6].

Plant-delivered antimicrobial chemicals (phytochemicals) represent a structurally diverse group of substances divided into three major groups: phenolic compounds, terpenes, and alkaloids. These compounds have exhibited beneficial antioxidant, antibacterial, and antifungal activities [7,8,9], and combined with antibiotics can act as effective resistance-modifying agents [7,10]. Enhancement of antimicrobial effect via a combination of different phytochemicals or antibiotics is referred to as synergism [11,12]. This synergistic approach to antibiotic application also offers other significant benefits, as a reduction in antibiotic dosage is directly associated with a decrease in the side effects of these drugs [7]. Among antimicrobial phytocompounds, eugenol, thymol, and curcumin have been reported to exhibit potent antimicrobial activity [7,8,13,14,15]. The above-mentioned compounds also showed synergic antimicrobial reactions with each other and with antibiotics [7,14].

These combinations have shown enhanced effectiveness against a broad spectrum of bacteria, including methicillin-resistant *Staphylococcus aureus*, Gram-negative bacteria, and oral pathogens, making them promising candidates for medical applications and resistance-modifying agents [14,15,16,17,18,19].

The phytochemicals also possess, besides antimicrobial activity, significant antioxidant and anti-inflammatory properties [20]. Numerous studies on animal models conclude that a reduction in oxidative stress at the wound site activates the pro-healing and anti-inflammatory gene pathways, thus promoting healing processes [21].

Proper wound healing requires a suitable environment to promote regeneration. Therefore, dressing material plays a crucial role in the treatment of wounds [1,22]. Previous studies revealed that the unique properties of nanofibres such as large surface, microporosity, and possibility of direct incorporation of active substances make nanofibres an attractive tool for local wound applications [23]. These nanostructures meet several demands of ideal wound dressings: provide terminal isolation and protection against infection, allow the exchange of gases and fluids between the environment and injured tissue, and, by mimicking the extracellular matrix, promote cell growth and proliferation [1,24,25].

**Figure 1:**
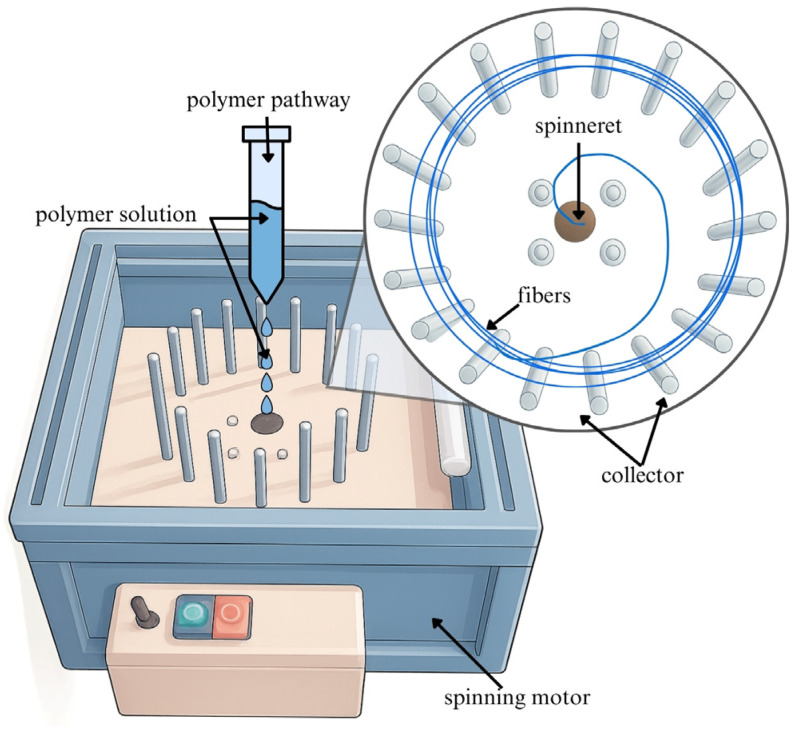
Schematic visualisation of centrifugal/forcespinning process.

Currently, a lot of different strategies for the fabrication of one-dimensional nanofibres from wide-scale polymers are available [23,26]. Centrifugally spun nanofibres (Figure 1) possess a porous structure that more accurately mimics the microenvironment of the extracellular matrix. Additionally, these nanofibres exhibit mechanical properties closely aligned with those of native tissues, making them promising candidates for wound dressings and other tissue-repair therapies [26]. To enhance healing processes, a vast number of different active substances have been incorporated into bioactive wound dressings and thus also into nanofibres, including antimicrobials, antibiotics, anti-inflammatory and anaesthetic drugs, growth factors, vitamins, minerals, and nano silver particles [25,27]. In this regard, several researchers started to focus on the incorporation of liposomes into nanofibres to utilise the combined benefits of both systems. In the development of hybrid systems, the literature provides two key strategies: coaxial and emulsion electrospinning methods [24,27,28,29,30]. Liposomal vesicles display high biocompatibility and biodegradability, low toxicity, and can be efficiently mass-produced. Additionally, these nanocarriers offer protection and enhancement of the stability and solubility of the cargo, improve transport through biological membranes, and allow targeted and controlled or prolonged release of incorporated substances [24,31,32]. Research conducted by Ternullo et al. presented a higher retention rate of conventional liposomes into the skin surface, confirming the depot effect of liposomes and their suitability for dermal drug delivery [32].

In some instances, different strategies involving immobilisation techniques have been employed to achieve a multifunctional integrated model [33,34,35]. However, to our best knowledge, no studies have reported the incorporation of liposomes into centrifugally spun poly(3-hydroxybutyrate) (PHB) nanofibres, or the utilisation of PHB-liposomes in such a system. PHB is categorised within the polyhydroxyalkanoates family and was utilised in the delivery system under investigation on the basis of its recognised high biocompatibility, biodegradability, and capacity for endotoxin-free production from renewable resources. The inherent properties of PHB have rendered it suitable for a variety of biomedical applications [36,37,38]. Moreover, our previously published research introduced new hybrid liposomal vehicles containing poly(3-hydroxybutyrate) (PHB-liposomes). The incorporation of the biopolymer improved the chemical stability of encapsulated phenolic compounds and the physiochemical stability of the liposomes. The presence of PHB in vehicles reduced toxicity and pro-inflammatory effects (IL-8) on the keratinocyte cell line compared to conventional liposomes [39,40].

The aim of the presented study was to develop an innovative type of delivery system based on a PHB-liposome-enriched nanofibrous mesh. Similar types of biologically active biomaterials containing PHB nanofibres and particle systems, with regard to short- and long-term release, are rarely found in the literature. To date, research has mostly focused on the application of PHB in the form of fibres [41,42,43,44] or films [45,46,47] and on PHB-liposomes separately [39,40,48]. However, the potential benefits of such a combined biologically active system have yet to be explored.

Centrifugal spinning was employed for the fabrication of PHB nanofibres, with PHB-liposomes immobilised within the nanostructure through adsorption. The objective was to integrate two different antimicrobials into PHB-liposomes and nanofibres, enabling their co-release at the wound site to enhance antimicrobial efficacy. This synergistic approach aims to reduce the likelihood of side effects while also addressing antibiotic resistance. Given the unique properties of both liposomes and nanofibres, this combined nanofibrous material shows promise as an advanced bioactive wound dressing [30].

## 2. Results

### 2.1. Antioxidant Activity

The presented research focused on natural compounds with significant biological activity that could be incorporated into skin delivery material. The over-production of oxygen species, and the subsequent oxidative stress that ensues, have been demonstrated to delay the healing process by prolonging the inflammatory phase and impairing cellular functions [49]. The antioxidant activity of thymol, eugenol, and curcumin is listed in Table 1.

In the case of selected natural compounds, there was no significant statistical difference between their antioxidant capacities. The *p*-values for these three samples were *p* < 0.05. All compounds showed high values of ABTS (2,2′-azino-bis(3-ethylbenzothiazoline-6-sulfonic acid) radical scavenging capacity in the range of 2.265–2.333 mmol TE/g, with the highest values exhibited by eugenol 2.333 ± 0.012 mmol TE/g.

### 2.2. Antimicrobial Susceptibility Test

The antimicrobial properties of natural compounds and antibiotics were tested against selected strains of Gram-positive and Gram-negative bacteria and yeast and evaluated by determination of minimal inhibitory and lethal concentrations (MICs and MLCs, respectively). Data are shown in Table 2. Prior to evaluation, the natural compound was dissolved in dimethyl sulfoxide (DMSO). For each microorganism, the potential antimicrobial activity of the solvent was evaluated. DMSO solutions were subsequently diluted by twofold serial dilution, and the concentration of solvent never exceeded the specified MIC. The variation between sample replicates and MIC and MLC were consistent between replicates.

Natural compounds (thymol, eugenol) showed MIC ranges of 0.06–4.00 mg/mL and MLC ranges of 0.13–8.00 mg/mL against Gram-positive bacteria. In the case of Gram-negative bacteria, the ranges were quite similar, starting at 0.10–2.00 mg/mL for MICs and at 0.2–4 mg/mL for MLCs. Bacterial strains were the least sensitive to curcumin, with MIC and MLC values exceeding 16 mg/mL in some selected strains. We were able to determine MIC and MLC values for curcumin of 0.06–16.00 mg/mL for *M. luteus*, *S. aureus*, *S. epidermidis*, and *P. aeruginosa*.

MICs for ampicillin were measured against Gram-positive bacteria, ranging from 0.4 to 0.6 µg/mL, while MLCs ranged from 0.8 to 2 µg/mL. The antibiotic showed an MIC of 6.25 µg/mL and MLC of 12.5 mg/mL against *E. coli.* The clinical bacterial strains were known to be resistant to ampicillin according to the literature [50,51,52,53], so no minimum inhibitory concentrations were measured. MICs for streptomycin against Gram-positive bacteria ranged between 6.30 and 200.00 µg/mL, with a decrease in antimicrobial effect against Gram-negative strains (3.10–8 µg/mL). MLCs for streptomycin ranged between 25.00 and 16 µg/mL against all bacteria. The selected strains were the most susceptible the to gentamicin, with MIC/MLC ranges of 0.02–0.90/0.5–12 µg/mL.

We also detected MICs for eugenol against *C. tropisaclis* and *C. parapsilosis* clinical strains at 1 mg/mL and the MLC at 2 mg/mL. The same minimum concentrations were obtained for *C. albicans* and *C. glabrata*, except for one sample of the *C. parapsilosis* strain where the MIC was found to be 0.5 mg/mL and the MLC 1 mg/mL.

The MIC and MLC for thymol were detected at the same values, 0.25/0.50 mg/mL, for *C. tropicalis* and all clinical strains of *C. parapsilosis*. The values decreased against *C. albicans* (0.06/0.13 mg/mL) and *C. glabrata* (0.13/0.25 mg/mL).

The highest minimum inhibitory and lethal concentrations against fungi were generally measured for curcumin. In some cases, we were not able to determine the specific values in all selected *Candida* strains, up to the upper limit of 16 mg/mL. For *C. tropicalis*, the MIC/MIL were established at 8/16 mg/mL, and for five clinical strains of *C. parabsilosis*, the MICs were found at concentrations of 1 mg/mL and 16 mg/mL. An MLC of 2 mg/mL was detected in two cases of these clinical strains. The lowest MIC/MLC values were measured against *C. albicans* at 0.13/0.25 mg/mL.

### 2.3. Synergistic Potential

The natural substances and antibiotics were investigated for their synergistic potential between each other and with antibiotics against all selected strains of bacteria and fungi. Only the most significant findings showing synergy or partial synergy are presented below in Table 3; all the other combinations showed indifferent results..

A synergistic effect with antibiotics was detected only with ampicillin (AMP), thymol, and eugenol. All the other combinations of tested compounds with antibiotics showed insignificant results. The measured synergistic potential with *S. epidermidis* (Fractional Inhibitory Concentration index (FICi) 0.32–0.35) decreased the MIC of ampicillin 128–16-fold, and another FICi in the range of synergism (FICi 0.50) was detected with thymol against *M. luteus.*

Thymol and eugenol are considered the most effective combination, showing synergism and partial synergism against *M. luteus*, *E. coli*, *S. epidermidis*, and *P. aeruginosa* clinical strains. The combination of natural compounds enhanced their antibacterial effect, and decreased the MICs of thymol and eugenol 2–4-fold. Partial synergy of this mixture was observed in all presented bacterial strains with measured synergy FICi values of 0.09–0.73.

### 2.4. Characterisation of PHB-Based Nanostructures

Hydrodynamic size and uniformity of liposomes in an environment of distilled water at room temperature were measured using dynamic light scattering. The data show the quite similar average size of PHB-liposomes loaded with active substances in the range of 151.90–199.20 nm.

The size distribution of loaded liposomes fluctuates around the value of particles without cargo (pure), 181.43 ± 7.80 nm. The uniformity of all suspensions was confirmed by low PDI values (0.19–0.23).

The liposome suspensions were tested for short- and long-term stability in a buffer environment at room temperature. The zeta potential of both time intervals indicated an ample negative charge amplitude, ensuring the stability of the liposomal suspension over a period of three weeks. Natural compounds had similar encapsulation efficiency in vehicles in the range of 90%. Ampicillin entrapment efficiency was 53.03 ± 0.04%. Liposomal diameter, polydispersity index, zeta potential, and entrapment efficiency are shown in Table 4.

The encapsulation efficiency of the forcespun nanofibres employed in the combined system was also the subject of evaluation (Table 4), with the highest value observed for eugenol (77.45 ± 5.63%).

### 2.5. Morphological Characterisation

**Figure 2:**
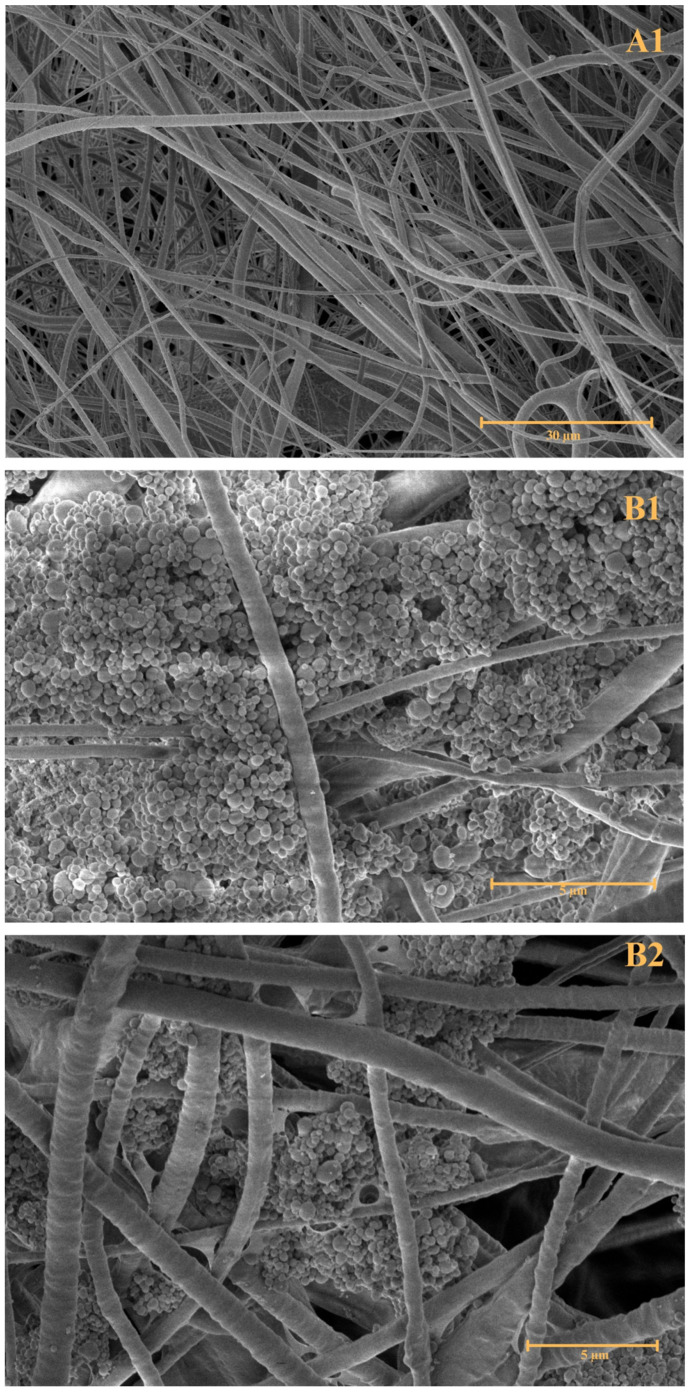
SEM images of the morphology of forcespun PHB fibres and combined structure; (**A1**) pure PHB nanofibres; (**B1**,**B2**) combined structures; (**A1**—magnification 3500×; **B1**—magnification 20,000×; **B2**—magnification 15,000×).

**Figure 3:**
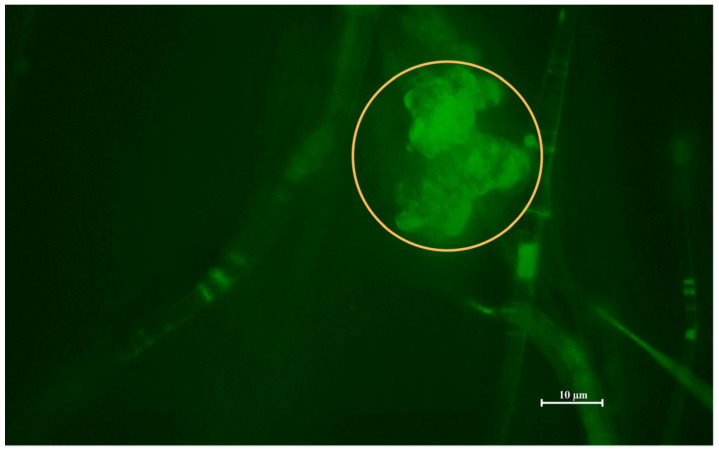
Image from the Fluorescence Microscope, fluorescent probe BODIPY^TM^, magnification 2000×. The cluster of PHB-liposomes is highlighted by the yellow circle.

To observe the morphology of the prepared combined nanofibre–liposomal delivery system, Scanning Electron Microscopy (SEM; Figure 2) and Fluorescence Microscopy (FM; Figure 3) were performed.

The first SEM image, A, showcases pure forcespun PHB nanofibres without any interference with their structural composition. The image A1, magnified 3500 times, supports the preparation of a bead-free nanofibrous structural system prepared by the forcespinning technique. The attachment of PHB-liposomal particles is predominantly believed to be based on adsorption interaction. The SEM images (B1–B2), magnified 20,000 and 15,000 times, show the individual particles attached in clusters between the gaps of fibres. The FM image (Figure 3) replicates the finding and marking of composite by the BODIPY^TM^ fluorescent probe, illuminating one of the liposomal clusters in the nanostructural mesh. The morphology of the cluster detected by FM correlates with the cluster base deposition of particles detected in images B1 and B2 by SEM. The combination of SEM and FM confirmed the successful incorporation of PHB-liposomes into the nanofibre mesh.

### 2.6. In Vitro Release of Encapsulated Substances

**Figure 4:**
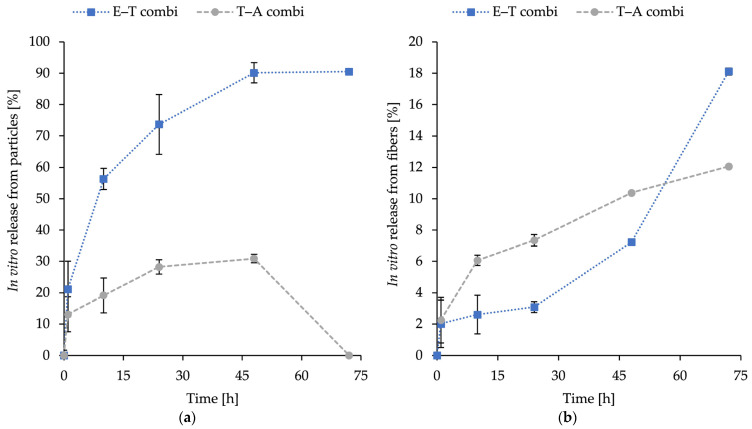
In vitro release of substances from immobilised liposomes (**a**) and nanofibres (**b**) from combined nanomaterial over 72 h in a 37 °C buffer environment. Results are presented as mean ± SD of *n* = 3; (E–T combi): eugenol in fibres and thymol in particles; (T–A combi): thymol in fibres and ampicillin in particles.

The release profiles of encapsulated substances inside combined structures—thymol, eugenol, and AMP, with E–T combi representing eugenol in fibres and thymol in liposomes and T–A combi representing encapsulated thymol in fibres and AMP in particles—are illustrated in Figure 4. The combinations were selected based on the synergistic potential measured in Table 3 and subsequently encapsulated in the dual-delivery system structure.

The percentage of release was calculated after 1, 10, 24, 48, and 72 h. The release was measured at 37 °C in PBS (pH 7.4) under shaking conditions. The objective was to evaluate the release potential of the novel fabricated dual nanostructure to assess the potential applications. The release profile obtained by this method represents a cumulative release of loaded compounds (thymol, eugenol, and ampicillin) up to each time point, assuming consistent behaviour among samples. The obtained release curves were subsequently analysed using standard kinetic models, with the understanding that each time point corresponds to an independent specimen.

The incorporation of particles resulted in elevated levels of released substances. For ampicillin, an initial release of 13.1 ± 1.7% at 1 h was observed, followed by a gradual increase to a maximum of 28.9 ± 2.3% at 48 h. The 72 h specimen was not found to contain any detectable ampicillin and was therefore excluded from kinetic fitting (see Section 3). The kinetic fitting of the ampicillin data was indicative of the Korsmeyer–Peppas model (R2 = 0.934) with an exponent *n* = 0.22, which is less than 0.45. This indicates that diffusion is the dominant mechanism. For thymol release from particles, a rapid release of 21.2 ± 7.6% was observed at 1 h and 56.3 ± 8.9% at 10 h. The rate of release continued to increase to 90.1 ± 9.5% at 48 h and then plateaued until it reached 90.5 ± 3.2% at 72 h, suggesting that a near-complete release occurred within 48 h. The thymol release kinetic model was found to be a good fit for both first-order (R2 = 0.970) and Korsmeyer–Peppas (R2 = 0.965) models. This suggests that the release process is concentration-dependent and involves a contribution from diffusion.

In addition, the release of eugenol and thymol from PHB-forcespun fibres was measured in the same physiological conditions as the release of the liposomal counterparts in dual systems. The release profile of both compounds was sustained, with initial release values recorded during the first 24 h, followed by a gradual increase. After 72 h, the maximum release levels were as follows: 12.44 ± 0.61% for thymol and 18.11 ± 0.23% for eugenol. The most effective release mechanism for eugenol was identified as the Weibull model (R2 = 0.9168), suggesting the involvement of degradation/erosion of the polymer matrix above diffusion. In contrast, the release of thymol followed the Korsmeyer–Peppas model most closely (R2 = 0.991), with *n* = 0.38, suggesting the primary release mechanism for diffusion.

### 2.7. Cytotoxicity of Composite Material

**Figure 5:**
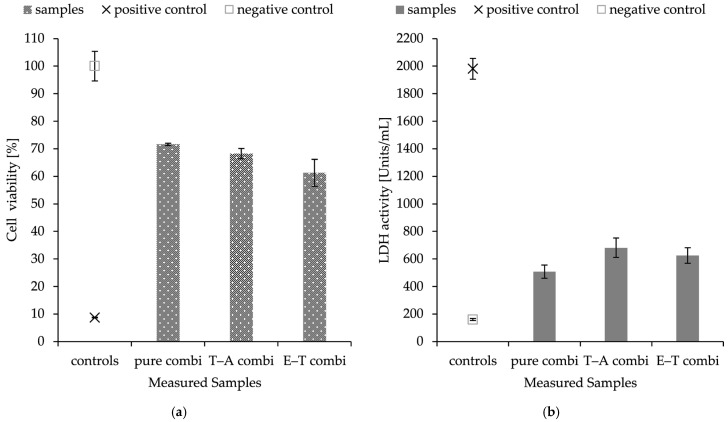
Cytotoxicity of HaCaT keratinocytes determined by MTT (**a**) and LDH (**b**) assays presented as mean with their respective SDs. Data represent two independent cell passages, each performed in triplicate (*n* = 6). The control values represent negative control with no samples affecting cells (MTT: 100.0 ± 5.4%; LDH: 159.7 ± 7.7 units/mL) and positive control with addition of DMSO to assure cell death (MTT: 8.7 ± 0.1%; LDH: 1980.8 ± 75.0 units/mL). Results are expressed as a percentage of the control; (pure combi): nanofibres with immobilised PHB-liposomes without encapsulated cargo; (E–T combi): eugenol in fibres and thymol in particles; (T–A combi): thymol in fibres and ampicillin in particles.

The objective of the assays was to determine the effect of prepared, functionalised materials on the viability of keratinocytes, and to assess the possible application of these materials in wound healing. The toxicity of the prepared nanostructures was evaluated after 24 h of exposure using two methods: the MTT assay, which assesses the metabolic activity of cells, and the lactate dehydrogenase (LDH) assay, measuring the activity of lactate dehydrogenase released from cells with compromised cell permeability, with the results shown in Figure 5.

HaCaT cells were exposed to combined delivery systems consisting of nanofibres and PHB-liposomes, each loaded with different substances. The results of the LDH assay and MTT assay are in accordance with each other. The materials showed cell viability above 50% in all cases. Both experiments revealed that the encapsulation of antimicrobial substances and the combinations of thymol–ampicillin and eugenol–thymol have the same effect on the viability of keratinocytes; pure combined material without any cargo scored similar values. The assays showed a decrease in metabolic activity and cell permeability with averages of 35.3 ± 3.4% and 29.5 ± 9.7% for combined structures.

### 2.8. Production of Inflammation Markers IL-6 and IL-8

**Figure 6:**
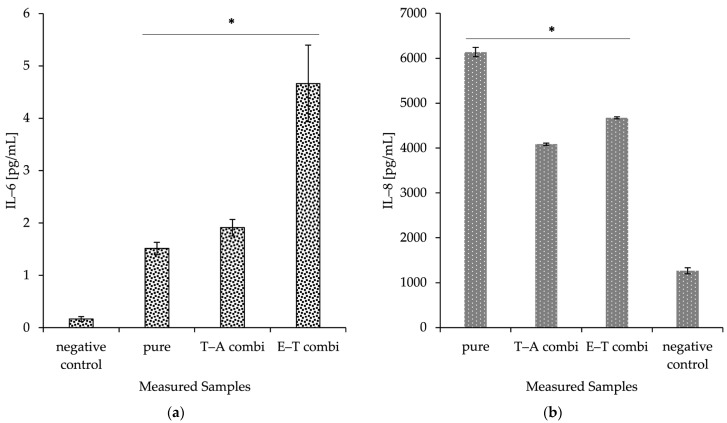
Anti-inflammatory activity represented as mean and SD (*n* = 3) of IL-6 (**a**) and IL-8 (**b**) proteins released from HaCaT cells exposed to nanofibres and combined nanomaterials; (pure): nanofibres without encapsulated cargo; (T): nanofibres with encapsulated thymol; (E): nanofibres with encapsulated eugenol; (E–T combi): eugenol in fibres and thymol in particles; (T–A combi): thymol in fibres and ampicillin in particles. Negative control represents the well with cells only treated by the culture medium. The IL-8 measurements were validated using the included quality controls, and the values fell within the specified control ranges. The asterisks indicate significant differences compared to negative control cells treated only with culture medium of *p* < 0.05, respectively.

As a preliminary immune safety screening, the composite delivery systems were evaluated for their potential to initiate IL-6 and IL-8 production in human dermal keratinocytes. The materials were observed to induce a significant increase in IL-6 and IL-8 protein production compared to controls. The prepared nanomaterials were also presented with nanofibres without any incorporated cargo (pure). This basic screening experiment suggests that the application of prepared nanofibrous mesh could stimulate the immune responses and thereby lead to an acceleration of primal healing processes. All the discussed data are visualised in Figure 6.

### 2.9. The Scratch Wound Healing Assay

The proliferation of the HaCaT cell line was not inhibited; therefore, the assay primarily assessed the re-epithelialisation process from the perspective of keratinocyte migration and proliferation. The wound model was created by introducing scratches into the cell monolayer using a sterile pipette tip. The process of re-epithelialisation was meticulously monitored by measuring the scratch width at baseline (0 h, immediately after scratching) and at two subsequent time points following the exposure of keratinocytes to the nanostructured samples.

**Figure 7:**
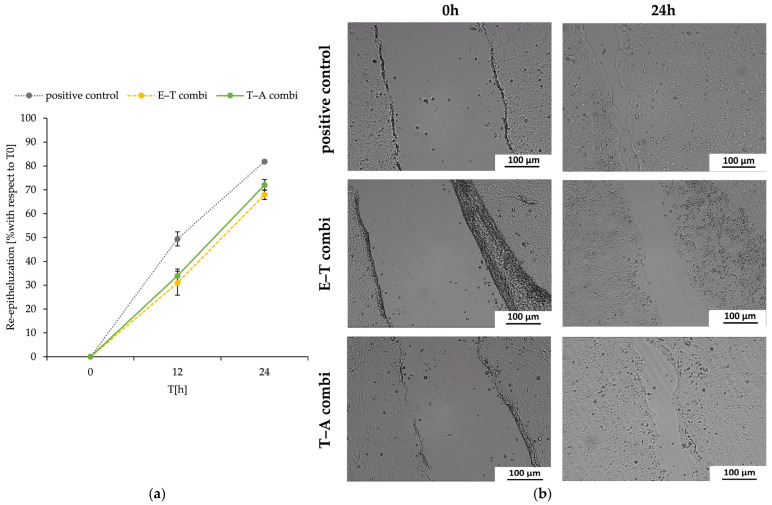
The representation of the re-epithelialisation process as a percentage of wound closure of the re-epithelialized area at time intervals of 0, 12, and 24 h (**a**) and side-by-side images (**b**) of a measured time point (0, 12, 24 h). Scale bars (100 µm) are included on the right-hand side of each image. The negative controls do not demonstrate any signs of wound closure, and therefore they cannot be quantified (values are not shown). Positive control represents the well with cells only treated by culture medium. The data (**a**) are presented as triplicates (*n* = 3) with ± SD. Subsequently, we evaluated the potential healing activity of the dual-delivery systems using the scratch assay, an established in vitro model of wound healing. The investigation focused on the regenerative potential of the combined systems containing encapsulated synergistic compounds, with the aim of inducing re-epithelialisation in human dermal keratinocytes, compared with the untreated negative control.

The findings, showed at Figure 7, demonstrated that treatment with the dual-delivery systems post-treatment did not result in a statistically significant decrease or increase in the re-epithelialisation rate of dermal keratinocytes when compared with the positive control.

### 2.10. Antimicrobial Activity of Combined Material

**Figure 8:**
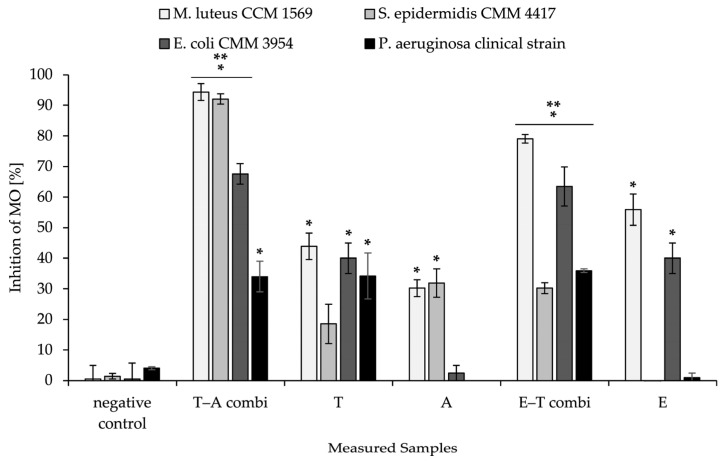
Antimicrobial activity of functionalised combined nanomaterials. The results are presented as mean ± SD from triple repetition of measurement; (MO): microorganism; (E–T combi): eugenol in fibres and thymol in particles; (T–A combi): thymol in fibres and ampicillin in particles; (T): nanofibres with encapsulated thymol; (E): nanofibres with encapsulated eugenol; (A): PHB-liposomes with encapsulated ampicillin. The positive control (cells treated with DMSO) was measured for all bacterial strains, with the average value being 102.63 ± 0.19% inhibition of MO growth. The asterisks indicate a significant difference compared to negative control cells treated with unfunctionalised nanostructures (*p* < 0.05). The double asterisks indicate a significant difference (*p* < 0.05) between the nanofibres loaded with an antimicrobial compound without the incorporation of a synergistic counterpart in liposomes and the dual fibre–liposome composite material.

The combined delivery system was also tested for antibacterial activity against selected strains of bacteria. The results are summarised in Figure 8. The Gram-positive and Gram-negative bacterial representatives were selected based on the synergistic potential of bioactive substances against them (Table 3).

In all samples of nanomaterial that were functionalised by antimicrobial substances, a significant decrease in bacterial growth was observed in comparison to the negative control, which consisted of nanofibrous mesh and PHB-liposomes without any loaded substances. The most significant results were achieved with a nanostructure consisting of nanofibres enriched by thymol, with immobilised liposomal particles loaded with ampicillin against Gram-positive bacteria in the tests. The results showed 94.34 ± 3.89% against *M. luteus* and 92.04 ± 1.70% against *S. epidermidis*. In the measurement, the most significant increase in antimicrobial activity was detected for the T–A combi compared to nanofibres loaded only with thymol against Gram-positive strains of bacteria (50.45% *M. luteus* and 73.56% *S. epidermidis*). Similarly, the T–A combi showed increased microbial inhibition against both Gram-positive and Gram-negative bacteria compared to particles loaded with ampicillin alone (60.15–65.15%). We detected the highest antimicrobial efficacy for both dual-delivery systems for the T–A combi at 94.34 ± 2.75% against *M. luteus.*

In addition, the inhibition of bacteria also increased significantly for the nanofibre–PHB-liposome combination of thymol and eugenol compared to purely eugenol nanofibres. This was observed against both selected Gram-positive and Gram-negative bacteria strains, with an average increase of 28%. The highest inhibition rate for the E–T combi was measured against *M. luteus* at 79.03 ± 1.96%. The nanofibre–PHB-liposome thymol–eugenol demonstrated increases in inhibition rate ranging from 23.15% to 34.90%, in comparison to thymol nanofibres devoid of particles. In a similar manner, the antimicrobial activity against the clinical strain of *P. aeruginosa* increased the most significantly from 1.0 ± 1.4% to 35.9 ± 0.6% following the incorporation of particles containing thymol into the eugenol fibrous mesh.

## 3. Discussion

Antioxidative activity has recently become a subject of increased research in the domain of wound treatment. An imbalance of free radicals and reactive species at the wound site has been demonstrated to induce oxidative stress, which in turn has been shown to prolong the inflammatory phase by sustaining pro-inflammatory cytokine signalling. Furthermore, this imbalance has been demonstrated to trigger the apoptosis of cells and promote the expansion of pathogens, thereby further delaying wound closure [49,54]. It has been demonstrated that the local neutralisation of harmful free radicals and reactive species at the wounded site by natural antioxidant compounds reduces redox imbalance and promotes wound healing pathways [21]. The selected natural substances are in most cases the primary compositional parts of the essential oils of plants, such as clove buds, common oregano, and thyme [55]. In isolation or as constituents of extracts and/or essential oils, curcumin, thymol, and eugenol are uniformly characterised as potent antioxidants across diverse studies [55,56,57,58,59]. The findings presented in Table 1 demonstrate similar antioxidant potential between the natural compounds, with values ranging from 2.333 to 2.265 mmol TE/g. A comparison of results across studies reveals comparable findings regarding the radical scavenging of thymol (2.341 ± 0.009 mmol TE/g) [60]. In the instance of eugenol, our findings demonstrate higher values in comparison to the study conducted by Adefegha et al., which reported a measurement of 2.58 µmol TE/g [61].

Normal skin microbiota is essential for the development and regulation of cutaneous immune function. However, even minor disruptions to the skin barrier can result in various clinical changes, including skin inflammation, infections, allergic reactions, tumour formation in the epidermis, and impaired wound healing [62].

The tested microorganisms were selected based on a literature search which identified the most significant wound pathogens that lead to infections and impaled healing of the wound bed. The studies identified a prevalence of Gram-positive streptococci, enterococci, and staphylococci species, and Gram-negative strains such as *Pseudomonas aeruginosa*, *Escherichia coli*, *Klebsiella* species, *Proteus* species, *Bacteroides*, and *Clostridium* spp. Along with bacteria, fungi are also classified as wound pathogens, with *Candida*, *Aspergillus*, and *Trichophyton* spp. being the most predominant contaminants [63,64]. In this context, we investigated the antimicrobial and synergistic effects of three natural compounds (thymol, eugenol, and curcumin) and the antibiotics (ampicillin, streptomycin, and gentamicin). Plant-derived bioactive compounds are widely considered not to contribute to the development of resistance, while exhibiting antimicrobial properties with rapid onset of action [65]. The natural compounds showed MICs (Table 2) in the range of 0.06–4.00 mg/mL against Gram-positive bacteria, 0.10–16.00 mg/mL against Gram-negative bacterial strains, and a range of MICs of 0.13–16.00 mg/mL against *Candida* spp. The present study also revealed predominantly higher resistance of Gram-negative bacteria in comparison to Gram-positive bacteria [65]. The peptidoglycan cell wall of Gram-positive cells permits easier penetration of hydrophobic molecules, causing the disruption of the bacterial structure [66]. The MIC of gentamicin was the lowest of all the antibiotics tested (0.02 µg/mL) against the Gram-positive *M. luteus* (Table 2). The antibacterial mechanism of the selected natural compounds is based on two main effects, damaging the structural integrity of cell membrane and, secondly, inhibiting enzymes responsible for energy-yielding processes [65,67]. The literature indicates these hydrophobic substances inhibit yeast growth through binding to ergosterol in the membrane, resulting in membrane destabilisation and eventual cell lysis [67,68].

The investigation revealed that certain compounds manifested not only antimicrobial activity but also synergistic antimicrobial potentiation and antibiotic-enhancing effects (Table 3). The combinations of thymol, eugenol, and ampicillin exhibited the most promising results, with FICi values indicating synergism and/or partial synergism against *S. epidermidis*, *M. luteus*, and *E. coli* and clinical strains of *P. aeruginosa*. The most significant effects were observed in combinations that reduced MIC values by at least twofold, particularly thymol with eugenol and thymol with ampicillin. The presented synergistic potential of natural compounds applied on their own and simultaneously with penicillin-like antibiotics is supported by the published literature [14,16,18,19,69]. For example, Moon et al. concluded that clove oil and its major component, eugenol, have a strong antibacterial effect against both cariogenic and periodontopathogenic bacteria, either on their own or in combination with antibiotics, thereby supporting the utilisation of clove oil/eugenol–antibiotic combinations for the treatment of oral infections [69].

Lipid-based nanostructures are well known and used in nanomedicine [24]. The present study focuses on the incorporation of liposomes into nanofibres to utilise the combined benefits of both systems. Primary particle characterisation (Table 4) revealed a similar average size, uniformity, and good long-term stability across all vesicles. The encapsulation efficiency of natural lipophilic compounds was found to be higher, around 90%, in comparison to ampicillin, which exhibited an encapsulation efficiency of 53.03 ± 0.04%. These findings are consistent with previous reports, which indicate that the water-repellent properties of hydrophobic substances enable more effective integration into liposomal structures compared to hydrophilic substances, resulting in higher encapsulation efficiencies [70].

Currently, a lot of different approaches to the production of nanofibres with higher yield rates at a larger scale are being investigated [23,26]. Centrifugal spinning processes are becoming more attractive due to the low-cost fabrication and high production efficiency [26]. The fabrication method has the potential to be employed as a tool to obtain a large number of micropores within the mesh, simultaneously with a bead-free feature, that could support the immobilisation of PHB-liposomes [26]. Current studies predominantly investigate the inclusion of secondary nanocarriers into nanofibre mats to ensure sustained controlled release [24,27,29,30,71]. Ding et al. investigated the possible usage of nanocomposite membranes to accelerate diabetic wound healing. A polyvinyl alcohol and chitosan nanofibre mesh with embedded taxifolin liposomes was created by emulsion spinning and biological experiments concluded that it stimulated the immune pathway and related pro-inflammatory factor, thus promoting the wound healing rate in mice [72]. A study by Hasanbegloo et al. also investigated liposome-loaded core–shell nanofibres for the treatment of breast cancer [73]. Nevertheless, to the best of our knowledge, no published studies have investigated the inclusion of PHB-liposomes into a PHB-nanofibre-based structure, which has been the motivation behind the execution of the present study. The morphology of PHB-liposomes was not shown in the current results, as the structure of these liposomes was described in our previous article (Bokrova et al). The particles were visualised by cryo-TEM microscopy and demonstrated the incorporation of polyhydroxyalkanoate. The PHB is covered by a layer of phospholipids and creates the multivesicular and multilamellar liposomal system [39]. The microscopy images presented in this article (Figure 2 and Figure 3) provide irrefutable confirmation of the successful preparation of a novel combined delivery system and the immobilisation of PHB-liposomes within a PHB-nanofibre mesh.

The decision to select the ideal nanopart of the combined structure for the incorporation of substances was also based on synergistic measurement (Table 3), where we also detected a higher amount of one of the two substances to obtain the synergistic effect. The substances with higher values measured from the checkerboard test were encapsulated into fibres and their counterparts into PHB-liposomes.

The dual release systems based on PHB-liposomes and PHB-nanofibres exhibited distinct release behaviour for particles and fibres (Figure 4). Ampicillin released from liposomes (from T–A combi) showed an initial release of 13.1% at the first hour, followed by a slow diffusion-limited release phase, stabilising at around 30% by 48 h. The undetectable release at the 72 h time point is likely indicative of the instability of the antibiotic in the aqueous buffer and therefore was excluded from kinetic fitting. β-lactam antibiotics are reported to undergo rapid hydrolytic degradation at physiological temperature, which we suspect as the primary course of action [74]. In contrast, thymol released from liposomes (E–T combi) exhibited rapid and almost complete release, reaching approximately 90% within 48 h. The release for both encapsulated substances from particles is similar, and both follow a sustained release profile as the release continues for more than 4 h, despite the initial burst release [75].

The release of loaded substances from fibres demonstrated sustained gradually increasing release, with both cases of the T–A combi and E–T combi reaching the maximum release of 12% and 18% at the 72 h time point. The measured data correspond with the research of Kundrat et al. [41], where the release of entrapped substances from PHB electrospun nanofibres at time intervals between 48 and 170 h was 12.5–20%, depending on the loaded substance. The difference in release rate between thymol and eugenol can be attributed to eugenol’s stronger specific interactions with the PHB matrix due to an additional methoxy group in the structure. The literature suggests the retained release and slower diffusion of compounds with stronger interactions to the entrapping polymer matrix [76,77]. The initial release, followed by the gradual increase in the release of phytochemicals, suggests that the release is influenced by the degradation or erosion of the polymer matrix [77]. The research by Kundrat et al. on drug release on PHB electrospun nanofibres showed similar findings [41].

The different release behaviours—slower, gradually increasing release for fibre-loaded substances, and the more rapid and (in the case of the E–T combi) nearly total release of substances from liposomes—suggest a complementary delivery potential of PHB-liposomal carriers in combination with PHB-nanofibres.

Following the incorporation of active substances with measured synergistic potential into the combined structures with PHB fibres and particles, the material exhibited increased bacterial inhibition in comparison to non-combined structures (Figure 8). The most notable increases in activity were observed against *S. epidermidis* for the T–A combi and against *P. aeruginosa* for the E–T combi. The measured slight increase in activity considering the negative control could be caused by the entrapment of microorganisms on the surface of the nanostructure before their removal from the well [78]. The inhibition value of the positive control that exceeded 100% could be explained by the control exerting a bactericidal effect in addition to merely inhibiting growth, and therefore reducing the viable cell count of the tested microorganisms to below the initial inoculum [79]. The finding supports our hypothesis of utilising the synergistic effect measured above to enhance the antibacterial potential of the nanofibrous delivery system.

The combined delivery system was created as a promising therapeutic agent in wound healing. The spontaneously immortalised human keratinocyte cell line (HaCaT) was utilised in the assessment of the material’s toxicity. HaCaT cells are considered highly suitable for the investigation of drug responses and immune mechanisms of keratinocytes and skin [80,81]. Together with toxicity and wound healing studies, the inflammation responses were investigated. The pro-inflammatory IL-8 and IL-6 cytokine production was used as an inflammation marker induced by combined materials. PHB-liposomes and pure PHB-nanofibres were studied in previously published articles [39,40,48]. The material showed a cell viability for the HaCaT cell line of 92.40 ± 4.20 %, supporting its non-cytotoxicity [48], and the incorporation of PHB into particles significantly reduced the pro-inflammatory effect of liposomes on the IL-8 chemokine [39]. Although the findings of the two cytotoxic assays indicated that the combination of materials caused a slight decrease in the metabolic activity of the HaCaT cell line (Figure 5), the re-epithelisation measurement in the scratch assay remained comparatively robust, with an average wound closure of 70% at 24 h for both dual-delivery systems (Figure 7). The measurement suggests that the nanostructured materials appear to suppress the metabolism of keratinocytes, though they do not fully impact the migratory and proliferation capacity of cells. These two systems have been shown to have significant antimicrobial, antioxidant, and immunological properties, making them promising candidates for future applications in local skin treatment.

## 4. Materials and Methods

### 4.1. Encapsulated Compounds

Natural compounds were purchased from Sigma-Aldrich (St. Louis, MO, USA): Curcumin, Eugenol, and Thymol. Ampicillin (Sigma-Aldrich, St. Louis, MO, USA) was used as the representative of antibiotics.

### 4.2. Antioxidant Activity

The antioxidant activity was evaluated using the Trolox equivalent antioxidant capacity (TEAC) assay with the use of pre-formed radical ABTS**·**+ mono-cation [82]. The ABTS radical was created by mixing 7 mM 2,2′-azino-bis(3-ethylbenzothiazoline-6-sulfonic acid) diammonium salt (Sigma-Aldrich, St. Louis, MO, USA) with 2.45 mM of potassium persulfate (Sigma-Aldrich, St. Louis, MO, USA) in deionized water, and the mixture was incubated in the dark at room temperature for 12–16 h. Before analysis, the ABTS**·**+ solution was diluted with ethanol to achieve an absorbance of A = 0.70 ± 0.02 measured spectrophotometrically at 734 nm. Then, 1 mL of the previously diluted ABTS**·**+ solution was mixed with 10 µL of the sample in an Eppendorf tube. The absorbance was recorded after 10 min of incubation of the sample in the dark. A calibration curve was constructed using Trolox dilutions within the concentration range of 40–400 µg/mL. Results are expressed as the mean ± standard deviation (SD) of three replicate determinations.

### 4.3. Antimicrobial Susceptibility

#### 4.3.1. Microbial Strains and Revitalization

Four microorganisms were acquired from the Czech Collection of Microorganisms (CCM, Brno, Czech Republic): *Micrococcus luteus* CCM 1569, *Streptococcus epidermidis* CCM 4417, *Escherichia coli* CCM 3954, and *Candida glabrata* CCM 8270.

Two strains of pathogenic bacteria and four strains of *Candida* spp. were isolated from different clinical samples at the Department of Biomedical Sciences, Section of Experimental and Clinical Microbiology, University of Sassari, Italy. The named microorganisms were cultivated and further worked with at the University of Sassari. The selected strains were a *Staphylococcus aureus* clinical strain, *Pseudomonas aeruginosa* clinical strain, *Klebsiella pneumoniae* clinical strain, 5 different isolates of *Candida parapsilosis* clinical strains 0–4, *Candida albicans* clinical strain, and *Candida tropicalis* clinical strain.

All cultures were stored in a cryoprotective medium at −80 °C. Before the experiment cultures were maintained on the appropriate media agar plate and cultured at 37 °C for 24 h. Prior to the experiment, inoculum was transferred to a liquid medium (LB broth (Luria Bertani Broth, Himedia, Thane, India), and BHI media (Brain Heart Infusion media, Himedia, Thane, India)) and cultivated on a shaker at 110–150 rpm and 37 °C for 24 h.

#### 4.3.2. Determination of Minimal Inhibitory Concentration (MIC) and Minimal Lethal Concentration (MLC)

The MIC and MLC of bacteria and fungi species were established by microdilution method using a 96-well microtiter plate as reported by the Clinical and Laboratory Standard Institute [83]. Lipophilic natural compounds were dissolved in dimethyl sulfoxide (DMSO, Sigma-Aldrich, St. Louis, MO, USA) and transferred to wells of the microplate. Afterwards, serial twofold dilution with LB broth (Luria Bertani Broth, Himedia, Thane, India), and BHI media (Brain Heart Infusion media, Himedia, Thane, India) was performed starting from 16 mg/mL for natural compounds and 25 µg/mL for ampicillin (Sigma Chemical Co., Burlington, MA, USA). The highest concentrations in the serial dilution contained 2.5% DMSO, which was confirmed in this study to have no effect on the viability of microbial cells. The 24 h inoculum grown was diluted in LB broth at a concentration of 0.5 McFarland, approximately 1.5 × 10^8^ cells/mL. The plates were prepared in duplicates at least and the experiments were repeated three times under the same conditions. Apart from the measured samples, the plate also contained controls of the sterility of media (MC), the sterility of the prepared sample (SC), and the complete inhibition of growth control (positive control) executed by 25% DMSO. A total of 100 µL of sample was added into each well together with 100 µL of bacterial/yeast suspension and incubated for 24 h. Minimal inhibitory concentration (MIC) is defined as the lowest concentration that still inhibits the growth of microorganisms in the ideal conditions supporting replication of microorganisms [84,85]. Minimal lethal concentration (MLC) was determined by transferring 10 µL from each well to the agar plate, prepared by the addition of 20 g/L of microbiological agar (Sigma-Aldrich, St. Louis, MO, USA) before sterilisation. Subsequently, agar plates were incubated for another 24 h at 37 °C. MLC is then determined visually as the minimal concentration of a substance that reduces the viability of culture and is standardly defined as a 99.9% kill rate [86]. Anaerobic microorganisms were placed into an anaerobic atmosphere during the duration of the experiments.

#### 4.3.3. Resazurin Reduction Assay

To assess the antimicrobial activity of prepared samples (also nanomaterials), resazurin sodium salt (Sigma-Aldrich, St. Louis, MO, USA) as an oxidation-reduction indicator was used for the visualisation of the metabolic activity of cells [87].

In the case of nanostructures, the assay was performed in a 24-well microplate. After 24 h incubation of culture with a sample of nanomaterial, 20 µL (96-well plate) and 100 µL (24-well microplate) of resazurin solution was added to the wells. The resazurin solution at a concentration of 0.15 mg/mL in PBS was sterilised by filtration (200 nm syringe filter, with polyethersulfone membranes). Afterwards, 0.5–3 h incubation at 37 °C followed, which continued until the development of colour. The colour change was recorded spectrophotometrically at an absorbance of 570 nm. Results are expressed as the mean ± standard deviation (SD) of three replicate determinations. During the calculations, the absorbance of wells only containing medium and resazurin was deducted from each well. The plates also contained controls: medium control, negative control (culture media with microorganisms or microorganisms treated by unfunctionalised nanostructures), and positive control (addition of 25% DMSO (Sigma-Aldrich, St. Louis, MO, USA)). Antimicrobial activity was calculated as a percentage of negative control (growth of microorganisms without interference) [87,88].

#### 4.3.4. Synergy Assay

The synergistic potential of natural compounds and antibiotic was evaluated using the microbial checkerboard microtiter assay. Twofold serial dilution of substance A was performed horizontally, followed by vertical twofold dilution of another compound, B. Each plate contained the MIC of the tested substance alone, medium sterility control, and growth control. Microorganisms were cultivated in 96-well plates and the culture was initially diluted to approximately 1.5 × 10^8^ cells/mL (0.5 McFarland or spectrophotometrically, as described above). To quantify the interaction between the compounds, the Fractional Inhibitory Concentration index values (FICi) were determined, following the equation: FIC_A_ + FIC_B_ = FICi.

The FIC_A_ is calculated by the deviation of MIC of compound A in combination with compound B by the MIC of compound A alone. FIC_B_ is defined in the same way, as the MIC of compound B in combination with compound B divided by the MIC of compound B. After 24 h incubation at 37 °C, the FICi value was assessed for each well with an ≥80% reduction in growth compared to the control. The FICi results were interpreted as synergy (FICi ≤ 0.5), partial synergy (FICi > 0.5 ≤ 1), indifference (FICi > 1 to <2), and antagonism (FICi ≥ 2) [41,42]. Results are obtained from triple replications of measurement with the same set of conditions.

### 4.4. Preparation of Nanostructures

#### 4.4.1. PHB-Liposome Preparation

Liposomes were formulated using a mixture of phosphatidylcholine (Sigma, St. Louis, MO, USA), cholesterol (Serva, Heidelberg, Germany, and 30% content of polyhydroxybutyrate (PHB, Hydal, Nafigate Co., Prague, Czech Republic). Antimicrobial substances were added at a concentration of 1 mg/mL. All necessary compounds were dissolved in chloroform and introduced into water. The resulting solution was sonicated for a duration of 60 s with a 13 mm diameter ultrasound probe (Sonopuls, Bandelin, Berlin, Germany). Thereafter the organic solvent was evaporated. Suspension of liposomes was lastly centrifugated at 11,000 rpm for 60 min. The supernatant was discarded, and the resulting nanoparticles were collected and resuspended in distilled water or buffer.

#### 4.4.2. Preparation of Nanofibres

Nanofibres were fabricated by the forcespinning technique when PHB (Hydal, Nafigate Co., Prague, Czech Republic) was dissolved in chloroform with antimicrobial substances added at a concentration of 5% (*w*/*w*) of nanofibre material. The polymer solution was slowly forcespun at 20–22 °C with a humidity of approximately 40–50%. After collecting nanofibrous mesh from the metal collector, the fibres were placed in Petri dishes and stored at laboratory temperature (natural compounds) and/or refrigerated (antibiotic).

#### 4.4.3. Preparation of Combined Structure

The combined material was prepared by placing forcespun nanofibres into a container with resuspended PHB-liposomes. The containers were shaken at 110 rpm (SHR-2D Shaker, Witeg, Wertheim am Main, Germany) for 24 h in the dark. After the time interval of exposure elapsed, the material was removed from solution and dried.

### 4.5. Characterisation of PHB-Based Nanostructures

#### 4.5.1. Encapsulation Efficiency

The encapsulation efficiency of particles was evaluated by measuring the solution after encapsulation. The samples were centrifugated for 60 min at 11,000 rpm (Z36 HK Centrifuge, Hermle, Gosheim, Germany), and afterwards the supernatant was filtered by a 0.45 µm filter, and the concentration of each remaining active compound was evaluated by HPLC. The specific analytical conditions are described in detail in the section describing the determination of the cumulative release of antimicrobial substances (Section 4.7).

The encapsulation efficiency of fibres was evaluated through the dissolution of nanofibre samples in 1 mL of chloroform. The solution was filtered through a 0.45 µm filter prior to UV-VIS determination of eugenol at 281 nm and thymol at 271 nm.

The encapsulation efficiency was calculated as the percentage of encapsulated substance to the total amount of substance added before encapsulation. Results are expressed as the mean ± standard deviation (SD) of three replicate determinations.

#### 4.5.2. Size and Distribution of Particles

The size of the liposomal nanoparticles was determined using dynamic light scattering (DLS). The particles were resuspended in distilled water and diluted 100-fold with distilled water prior to measurement. Each sample was measured in triplicate using a Malvern Zetasizer (Worcestershire, UK); from the obtained results, the average size of particles and distribution were calculated.

#### 4.5.3. Stability Assessment of Liposomes

To evaluate the stability of the prepared particles, zeta potential measurement was conducted with a Malvern Zetasizer. The liposomes were resuspended in PBS buffer (VWR^®^, Phosphate-buffered saline 1X, pH ≈ 7.4, Radnor, PA, USA) and prior to measurement, the samples were subsequently diluted 100-fold with buffer. The suspension was placed inside a dip cell and analysed. The short- and long-term stability was determined based on measured zeta potential. Short-term stability was initially measured at the 0 h time point, immediately after the preparation of liposomes. To assess long-term stability, the particles were, after resuspension in a buffer, left to rest for the duration of the stability assessment for 2 weeks at room temperature (approximately 25–26 °C). After incubation, the zeta potential was measured in triplicate, following the procedure described above.

### 4.6. Morphological Characterisation

Scanning electron microscopy was utilised for the characterisation of nanomaterial. SEM with a Versa 3D (ZEISS, Jena, Germany) microscope was operated in high-vacuum mode with an acceleration voltage of 2 kV. The sample surfaces were sputter-coated with a 5 nm layer of platinum. Fibre diameters were measured using the SmartSEM V6.08 software (ZEISS, Jena, Germany). The original, unmodified SEM images of the morphology of the forcespun PHB fibres and the combined fibre-liposome structures (A1; B1; B2) are provided in the Supplementary Materials (Figures S7–S9).

Simultaneously, the material was visualised by Fluorescence Microscopy (Nikon Eclipse E400, Nikon; Tokyo, Japan) and by a BODIPY^TM^ fluorescent probe (Thermo Fisher, Waltham, MA, USA). BODIPY is a small molecule of fluorescent dye that possesses low phototoxicity and photobleaching, strong absorption and emission bands, high quantum yield, and stable chemical structure [89]. The original, unmodified images from Fluorescence Microscopy are provided in the Supplementary Materials (Figure S10).

### 4.7. In Vitro Release of Encapsulated Substances

The release of antimicrobial substances from nanostructures was carried out by placing prepared combined structures inside an Eppendorf tube containing PBS buffer (VWR^®^, Phosphate-buffered saline 1X, pH ≈ 7.4, Solon, OH, USA) and incubating at conditions of 37 °C on a shaker at 110–150 rpm at for 1, 10, 24, 48, and 72 h. After conclusion of the incubation period, the PBS solution was filtered using a nylon filter with pores of 0.45 µm and diluted accordingly. The solution was subsequently analysed by HPLC (High-Performance Liquid Chromatography). A fresh sample of material was released at each time interval (*n* = 3). The solution was then analysed using HPLC. A new sample of material was required for release at each designated time interval. Therefore, each time point represents the total amount of compounds released up to that time.

HPLC analysis was conducted using an HPLC/PDA Dionex UltiMate 3000 (Thermo Fischer Scientific, Waltham, MA, USA) and Kinetex C18 column, with 150 mm length, 4.6 mm width, and 2.5 µm particle size. Different separation conditions of the mobile phase were applied to natural compounds (thymol, eugenol) and antibiotic ampicillin.

Natural compounds were separated by applying gradient elution of a mixture of mobile phase of 0.01% trifluorooctanoic acid (phase A, Honeywell Fluka^TM^, purity ≥ 99.0%, Seelze, Germany) and acetonitrile (phase B, HPLC grade ≥ 99.9%; Honeywell, Seelze, Germany). The total run time of the method was 20 min, at the conditions of 25 °C and flow rate of 0,4 mL/min. The concentration gradient was changed as follows: (a) initially 75% A and 25% B, (b) 5 min 60% A and 40% B, (c) 10 min 45% A and 55% B, (d) 12 min 30% A and 70% B, and (e) 20 min 75% A and 25% B. The UV-VIS spectra of individual natural compounds were analysed at a 280 nm wavelength.

The amount of ampicillin was measured using isocratic elution mobile phase mixtures A: 0.01% trifluorooctanoic acid (phase A, Honeywell Fluka^TM^, purity ≥ 99.0%, Seelze, Germany) and B: acetonitrile (phase B, HPLC grade ≥ 99.9%; Honeywell, Seelze, Germany), with the ratio 80:20. The total run time was 20 min, at conditions of 25 °C and a flow rate of 0.4 mL/min. The UV-VIS spectra were evaluated for wavelength of 210 nm.

The cumulative release was calculated as a percentage of released substance to the total amount of substance remaining encapsulated inside nanostructures after 24 h. The total amount was specified by dissolving combined nanostructures in a chloroform solution (Honeywell CHROMASOLV™, for HPLC, ≥99.8%, amylene stabilised, Seelze, Germany) and measured by the spectroscopic technique. The absorbance of natural compounds was detected at 281 nm for eugenol, and 275 nm for thymol. For the detection of total ampicillin, deionized water was added to the chloroform solution in a ratio of 1:1 and shaken. Afterwards, the water phase was removed and analysed by the HPLC procedure described above.

### 4.8. Cytotoxicity Assays

#### 4.8.1. MTT Assay

The HaCaT cell line was obtained from CLS Cell Lines Service GmbH, Eppelheim, Germany (Cat. No. 300493). The HaCaT keratinocyte cell line was cultured in Dulbecco’s Modified Eagle’s Medium (DMEM) supplemented with 10% heat-inactivated foetal bovine serum and 1% penicillin/streptomycin/amphotericin B mixture (Gibco, Waltham, MA, USA). The keratinocytes were cultivated in a humidified atmosphere of 5% CO_2_, at 37 °C (IPP110ecoplus Incubator, Memmert, Schwabach, Germany). Cells were seeded into 24-well flat-bottom microplates at a density of 25.10^3^ cells/well and allowed to adhere to the bottom of the plate and grow for 16–24 h. After incubation, the medium was refreshed, and the prepared nanomaterials were added into wells and incubated for 24 h. MTT (3-(4,5-dimethylthiazol-2-yl)-2,5-diphenyltetrazolium bromide; Duchefa) at a concentration of 2.5 mg/mL in PBS and sterilised by 200 nm syringe filter, with polyethersulfone membranes. After incubation, 20 µL per well of MTT was added and incubated for 3 h at 37 °C. The formazan crystals formed by the function of mitochondrial reductase were dissolved in a detergent solution and measured spectrophotometrically at 540 nm using a microplate reader (BioTek Epoch Microplate Spectrophotometer, Winooski, VT, USA). Prior to the experiments, all the prepared nanostructures had been sterilised by ultraviolet germicidal irradiation for a duration of 20 min. The solution of DMSO (Carl Roth GmbH + Co. KG, Karlsruhe, Germany) at a concentration of 15% served as positive control (kill control) and an unmodified medium was used as negative control (cell growth control). The data represent two independent cell passages, each performed in triplicate (*n* = 6). The viability of cells was calculated according to the formula: cell viability (%) = (absorbance of cells treated with prepared material (absorbance of cells treated with unchanged culture medium) × 100.

#### 4.8.2. LDH Assay

Lactate dehydrogenase (LDH) is an enzyme measured upon release from cell cytoplasm caused by damage to the cell membrane. The values of LDH were calculated using a standard curve of sodium pyruvate at a concentration equivalent to LDH activity of 0–2000 U/mL. The supernatant sample originated from the incubation of tested materials with HaCaT keratinocytes for 24 h in a humidified atmosphere of 5% CO_2_, at 37 °C (IPP110ecoplus Incubator, Memmert, Schwabach, Germany). The HaCaT cell line was obtained from CLS Cell Lines Service GmbH, Germany (Cat. No. 300493). The data are measured in absolute LDH activity units. The positive control samples, representing the maximum LDH release into the medium, were prepared by treating cells with 15% DMSO (Carl Roth GmbH + Co. KG, Karlsruhe, Germany). The negative control consisted of cells incubated in an unmodified medium only.

A 10 µL aliquot of the appropriate standards, supernatant sample, and controls was pipetted into a 96-well plate. The content of each well was repeated three times for at least two different cell passages and 50 µL of an NADH (β-nicotinamide-adenine disodium salt, VWR®, Lutterworth, Germany) solution in pyruvate (VWR^®^, Solon, OH, USA) was added to the microplate. After 30 min of incubation at 37 °C, 50 µL of a 2.4-DNPH (2.4-dinitrophenylhydrazine, Sigma-Aldrich, St. Louis, MO, USA) solution in 1M HCl (Hydrochloric acid, Lach-Ner, Neratovice, Czech Republic) was added to each well, and the plate was agitated at room temperature for 20 min in the dark. The reaction was stopped by the addition of 4M NaOH (50 µL) (Sodium hydroxide, Lach-Ner, Neratovice, Czech Republic) and the absorbance was measured at a wavelength of 540 nm, using a BioTek Epoch Microplate Spectrophotometer Winooski, VT, USA. The data represent two independent cell passages, each of which was performed in triplicate (*n* = 6).

### 4.9. Production of Inflammatory Markers IL-8, IL-6

Immune response assays were conducted using Invitrogen kits, specifically the Human IL-6 ELISA and Human IL-8 ELISA kits (Invitrogen, Waltham, MA, USA). Both assays were performed following the protocols outlined in the respective manuals: MAN0014630 (rev. 3) and MAN0018696 (rev. 1). The absorbance values were read using a BioTek Synergy HTX multimode reader, Winooski, VT, USA.

### 4.10. The Scratch Wound Healing Assay

Epidermal HaCaT keratinocytes were seeded at a density of 25.10^3^ cells/well in 24-well tissue culture microplates, with each treatment being replicated three times. Twenty-four hours after seeding the cells, the cell monolayer was scraped mechanically using a sterile 1000 µL pipette tip. Scratch assays were terminated at 24 h, a pre-specified endpoint based on pilot kinetics showing approximate 80–90% closure in positive controls (cells only treated by supplemented culture medium) without complete closure. The appropriate minimal media were added as negative controls. The HaCaT cell line was obtained from CLS Cell Lines Service GmbH, Germany (Cat. No. 300493).

UV light-sterilised (20 min) dual-composite materials were added to keratinocytes together with the culture media. Immediately after wound induction (T0), and at 12 and 24 h after treatment, the microplates were placed under the microscope with a digital camera to assess the wound conditions. The distance between the two sides of the wound area was measured using ToupView 3.7 software (Toupek Photonics, Hangzhou, China). For clarity, the software measurement overlays have been removed from the images shown. The original, unmodified images of re-epithelialisation process representing positive control A–T and E–T combi at time 0 h and 24 h are provided in the Supplementary Materials (Figures S1–S6).

To ensure the accuracy of the results, each wound site was measured at multiple locations (at least *n* = 3). The re-epithelisation process was measured by calculating the percentage of wound closure at various time points between 0 h and 24 h (mean ± standard deviation, *n* = 3 independent experiments, each in triplicate). The data were then analysed using Student’s *t*-test, which revealed significant results with a *p*-value less than 0.05.

### 4.11. Statistical Analysis

All experiments were conducted in multiple replicates (at least three in each experiment). Data are expressed as mean ± standard deviation (SD). Statistical analysis was performed using one-way ANOVA with a significance level set at 0.05 to test difference among groups. The pairwise comparisons of each sample between each other and vs. control were performed by a two-sample *t*-test. A value of *p* < 0.05 was considered significant. All statistical analyses were conducted in Microsoft Excel (Microsoft Office 365, Microsoft Corporation, Redmond, WA, USA)

## 5. Conclusions

The prepared composite material, incorporating encapsulated antimicrobial substances, shows potential for localised skin treatments. The selected natural compounds demonstrated potent antioxidant and antimicrobial activities against representatives of Gram-positive and Gram-negative bacteria, as well as fungi. The synergistic effect of thymol eugenol, when combined with each other and/or with ampicillin, was detected against *E. coli*, *S. epidermidis*, *M. luteus*, and *P. aeruginosa*. The incorporation of antimicrobial agents into liposomes maintained the small size and colloidal stability of particles. Microscopic analysis confirmed the successful preparation of the combined nanofibrous material containing liposomal particles in its gaps. Notably, two synergistic combinations, eugenol–thymol and thymol–ampicillin, exhibited promising biological properties after encapsulation into the newly prepared dual-nanostructured material. The combined nanomaterials demonstrated an enhancement in antibacterial activity. Although the slight decline in metabolic activity exhibited by the combined material in the HaCaT cell line, further evaluation is nevertheless possible, as the material could be characterised as not cytotoxic. Moreover, the utilisation of the material has been demonstrated to elicit immune responses, as evidenced by the elevated levels of inflammatory markers IL-6 and IL-8. In addition, the wound healing assay has revealed the re-epithelisation of the wounded area. The results suggest that the newly developed nanofibrous system could contribute to strategies aiming to reduce the usage of antibiotics and minimise the side effects associated with excessive doses of active substances. Besides its potent antimicrobial properties, the lipophilic natural compounds exhibited strong antioxidant effects, which may promote healing processes. The promising qualities of the prepared new nanofibrous system, coupled with the results of this study, support its potential for further development as a combined delivery system. Applied dermally, the material could be potentially utilised for the localised treatment of severe injuries, as well as for various cosmetic applications. However, further in vivo studies are needed to evaluate its clinical effectiveness.

## Figures and Tables

**Table 1:** Antioxidant activity of natural compounds measured by Trolox equivalent antioxidant capacity (TEAC) method. Values are represented as mean ± SD of tree replicates.

Sample	Antioxidant Activity [mmol TE/g]
Eugenol	2.333 ± 0.012
Thymol	2.265 ± 0.017
Curcumin	2.294 ± 0.003

**Table 2:** MIC/MLC of natural compounds in mg/mL and antibiotics in µg/mL. MIC/MLC values are reported with two decimal places to reflect the twofold serial dilution scheme. Experimental variability is represented by replicates (*n* = 3) and MIC and MLC were consistent between replicates. (MLC) minimal lethal concentration, (MIC) minimal inhibitory concentration, (AMP) ampicillin, (STR) streptomycin, (GEN) gentamicin.

Strains	Measured Compounds and Concentrations					
	Curcumin mg/mL	Thymol mg/mL	Eugenol mg/mL	AMP µg/mL	STR µg/mL	GEN µg/mL
**Gram-positive bacteria**						
*M. luteus* CCM 1569	>16.00/0.13	0.12/0.50	0.50/1.00	0.60/2.00	6.30/100.00	0.02/12.00
*S. aureus* clinical strain	0.06/0.13	0.06/0.13	0.50/1.00	nd ^1^	nd ^1^	nd ^1^
*S. epidermidis* CCM 4417	>16/2	0.50/2.00	4/8	0.40/0.80	200/400	0.10/0.50
**Gram-negative bacteria**						
*E. coli* CMM 3954	>16/>16	0.25/2.00	2/4	6.25/12.50	3.10/25.00	0.90/3.10
*P. aeruginosa* clinical strain	16/>16	0.40/0.75	1/2	nd ^1^	8/16	0.50/1.00
*K. pneumoniae* clinical strain	>16/>16	0.10/0.20	0.35/0.75	nd ^1^	8/16	2/1
**Yeast**						
*C. tropicalis* clinical strain	8/16	0.25/0.50	1/2	nd ^1^	nd ^1^	nd ^1^
*C. parapsilosis* clinical strain 0	16/>16	0.25/0.50	1/2	nd ^1^	nd ^1^	nd ^1^
*C. parapsilosis* clinical strain 1	1/2	0.25/0.50	0.50/1.00	nd ^1^	nd ^1^	nd ^1^
*C. parapsilosis* clinical strain 2	1/2	0.25/0.50	1/2	nd ^1^	nd ^1^	nd ^1^
*C. parapsilosis* clinical strain 3	16/>16	0.25/0.50	1/2	nd ^1^	nd ^1^	nd ^1^
*C. parapsilosis* clinical strain 4	16/>16	0.25/0.50	1/2	nd ^1^	nd ^1^	nd ^1^
*C. albicans* clinical strain	0.13/0.25	0.06/0.13	0.50/1.00	nd ^1^	nd ^1^	nd ^1^
*C. glabrata* CCM 8270	>16/>16	0.13/0.25	0.50/>16.00	nd ^1^	nd ^1^	nd ^1^

^1^ (nd) not detected.

**Table 3:** The significant synergism results of natural compounds and AMP; the values were determined from at least three independent experiments.

Compounds		FICi ^2^							
		*P. aeruginosa* Clinical Strain		*S. epidermidis*		*M. luteus*		*E. coli*	
Thymol	Eugenol	0.73	PSYN ^3^	0.09	SYN ^3^	0.69	PSYN ^3^	0.58	PSYN ^3^
AMP ^1^	Thymol	nd ^4^	IND ^3^	0.32	SYN ^3^	0.50	SYN ^3^	1.02	IND ^3^
	Eugenol	nd ^4^	IND ^3^	0.35	SYN ^3^	1.00	IND ^3^	1.03	IND ^3^

^1^ AMP: ampicillin, ^2^ FICi: Fractional Inhibitory Concentration index, ^3^ IND: indifference, PSYN: partial synergism, SYN: synergism, ^4^ (nd): not detected.

**Table 4:** Characterisation of PHB-liposomes and PHB-nanofibres with encapsulated compounds. Results are presented as mean ± SD of *n* = 3.

Sample Loaded Liposomes	Liposome Diameter [nm]	Liposomal PDI ^2^	Zeta Potential of Liposomes [mV]		Encapsulation Efficiency of Liposomes [%]	Encapsulation Efficiency of Fibres [%]
			Short-Term Stability	Long-Term Stability		
Pure	181.43 ± 7.80	0.20 ± 0.05	−33.00 ± 2.41	−21.77 ± 1.45	— ^1^	— ^1^
Thymol	199.20 ± 8.15	0.22 ± 0.02	−41.47 ± 2.66	−22.03 ± 1.16	90.05 ± 0.26	67.31 ± 8.34
Eugenol	151.90 ± 15.36	0.23 ± 0.08	−26.07 ± 2.14	−22.73 ± 2.02	90.00 ± 0.19	77.45 ± 5.63
Ampicillin	172.20 ± 3.46	0.19 ± 0.01	−36.16 ± 0.99	−30.00 ± 0.47	53.031 ± 0.04	— ^1^

^1^ (—) not tested, ^2^ (PDI) polydispersity index.

## Data Availability

The data presented in this study are available on request from the corresponding author.

## Supplementary Materials

The following supporting information can be downloaded at: https://www.mdpi.com/article/10.3390/ijms26199485/s1.

## Abbreviations

The following abbreviations are used in this manuscript:

PHB	Poly(3-hydroxybutyrate)
DMSO	Dimethyl sulfoxide
AMP	Ampicillin
STR	Streptomycin
Gen	Gentamicin
MIC	Minimal inhibitory concentration
MLC	Minimal lethal concentration
FICi	Fractional Inhibitory Concentration index values
IND	Indifference
PSYN	Partial synergism
SYN	Synergism
E	Eugenol
T	Thymol
E–T combi	Eugenol in fibres and thymol in particles combination
T–A combi	Thymol in fibres and ampicillin in particles combination
SEM	Scanning Electron Microscopy
FM	Fluorescence Microscopy
TEAC	Trolox equivalent antioxidant capacity
ABTS	2,2′-azino-bis(3-ethylbenzothiazoline-6-sulfonic acid)
TE	Trolox equivalent
CCM	Czech Collection of Microorganisms
LB	Luria–Bertani Broth
BHI	Brain Heart Infusion media
SD	Standard deviation
DLS	Dynamic light scattering
PBS	Phosphate-buffered saline
HPLC	High-Performance Liquid Chromatography
LDH	Lactate dehydrogenase
NADH	β-nicotinamide-adenine
2.4-DNPH	2.4-dinitrophenylhydrazine
HCl	Hydrochloric acid
NaOH	Sodium hydroxide
