# Supplementary figures and images for: Nanostructured Dual-Delivery System with Antioxidant and Synergistic Approach for Targeted Dermal Treatment
Source: Int J Mol Sci. 2025 Sep 28;26(19):9485. doi: 10.3390/ijms26199485 (PMC12525100; doi:10.3390/ijms26199485)

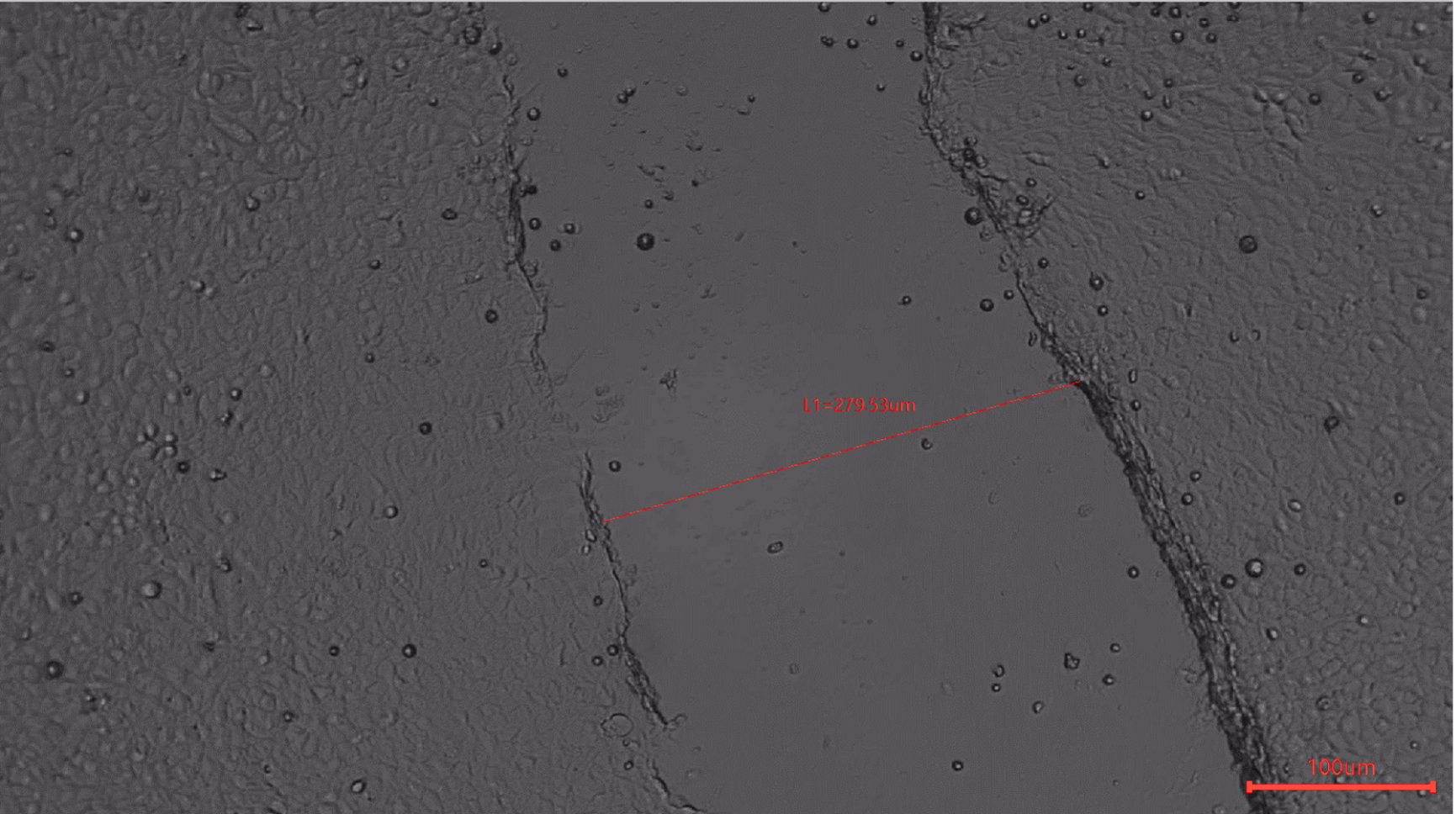

Supplement: Supplementary file 1 [file ijms-26-09485-s001.zip › Figure S1 Original image of the re-epithelialisation process representing A-T combi at time 0h.png]

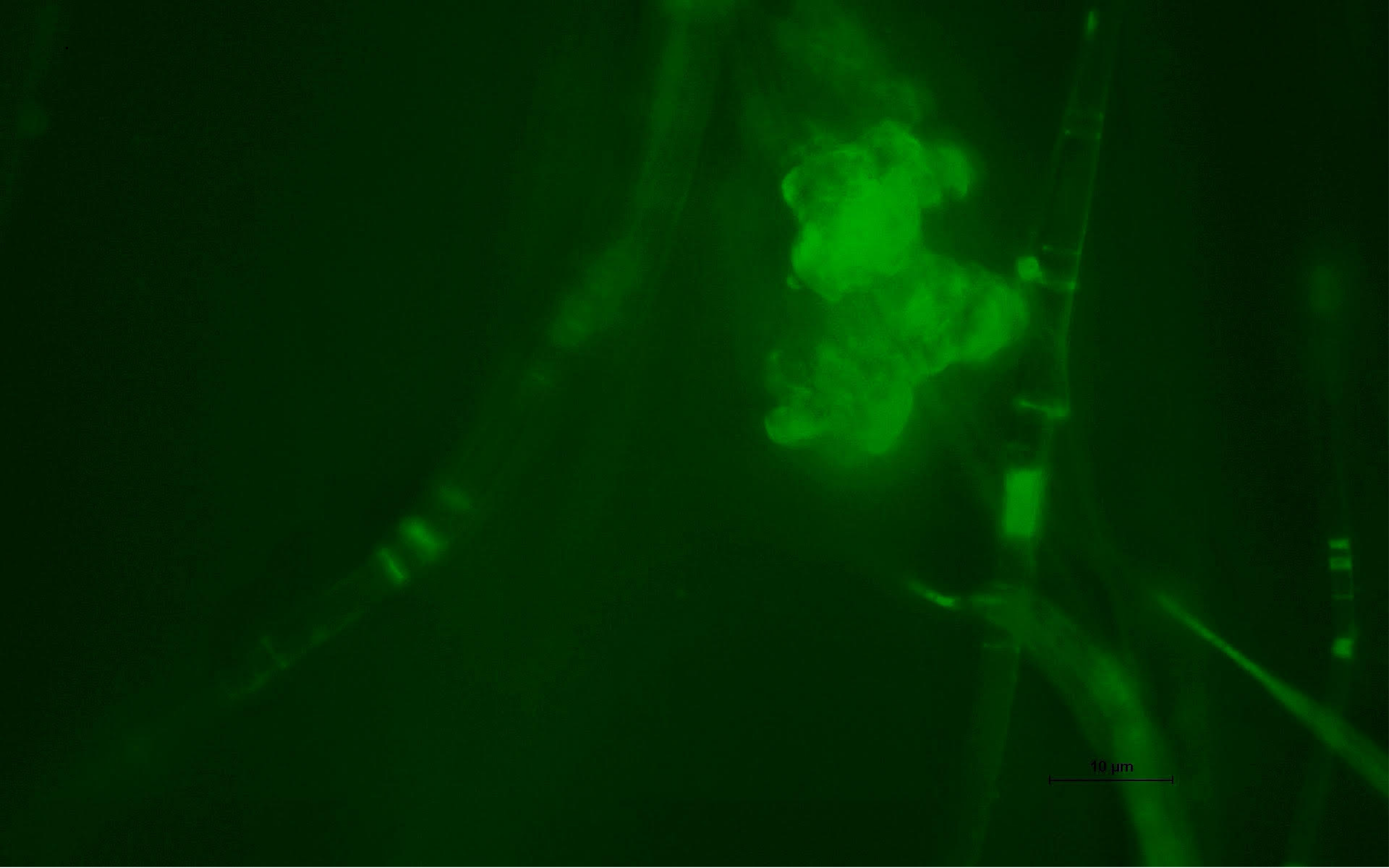

Supplement: Supplementary file 1 [file ijms-26-09485-s001.zip › Figure S10 Original image from the Fluorescence Microscopy.png]

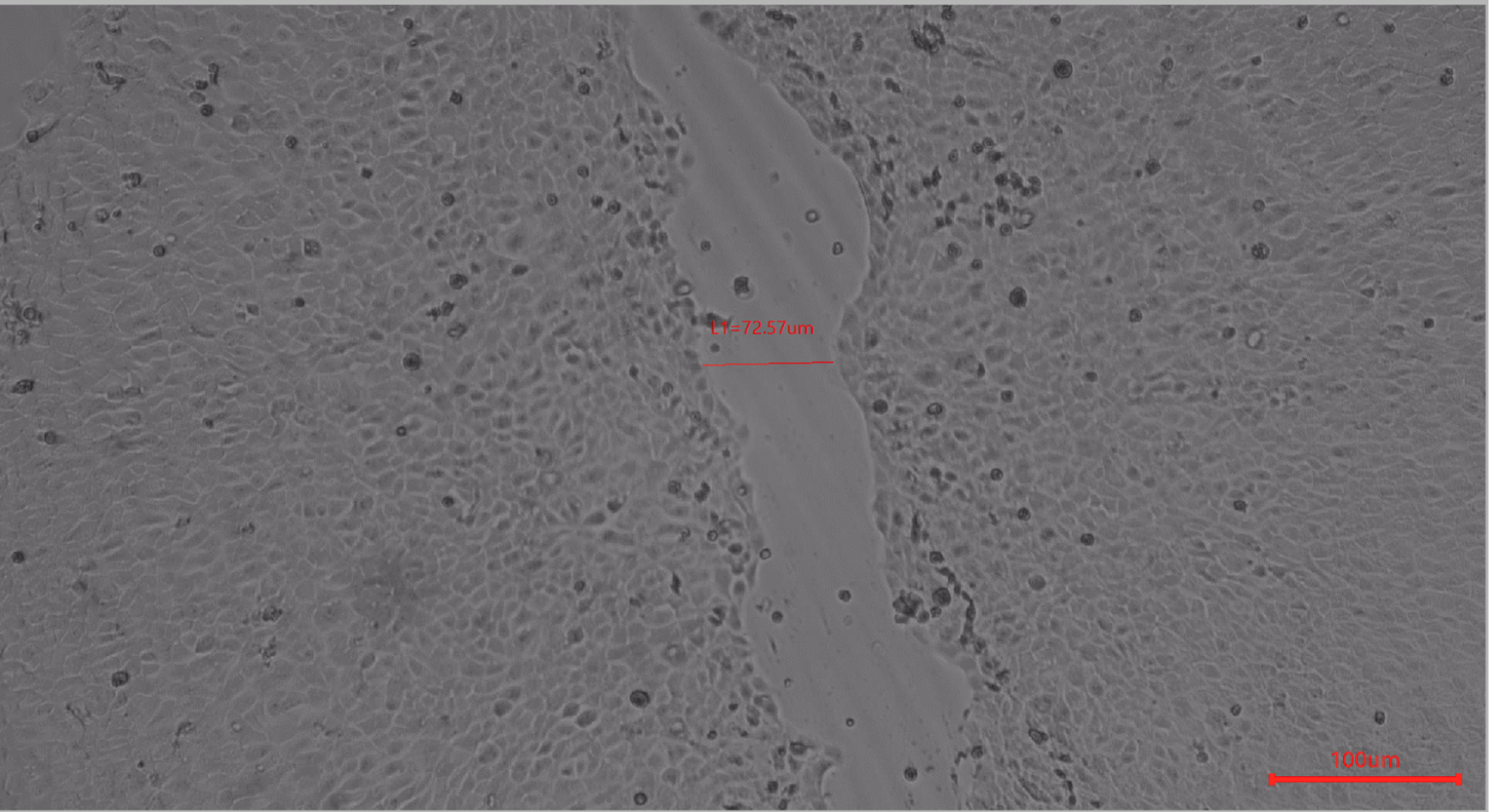

Supplement: Supplementary file 1 [file ijms-26-09485-s001.zip › Figure S2 Original image of the re-epithelialisation process representing A-T combi at time 24h.png]

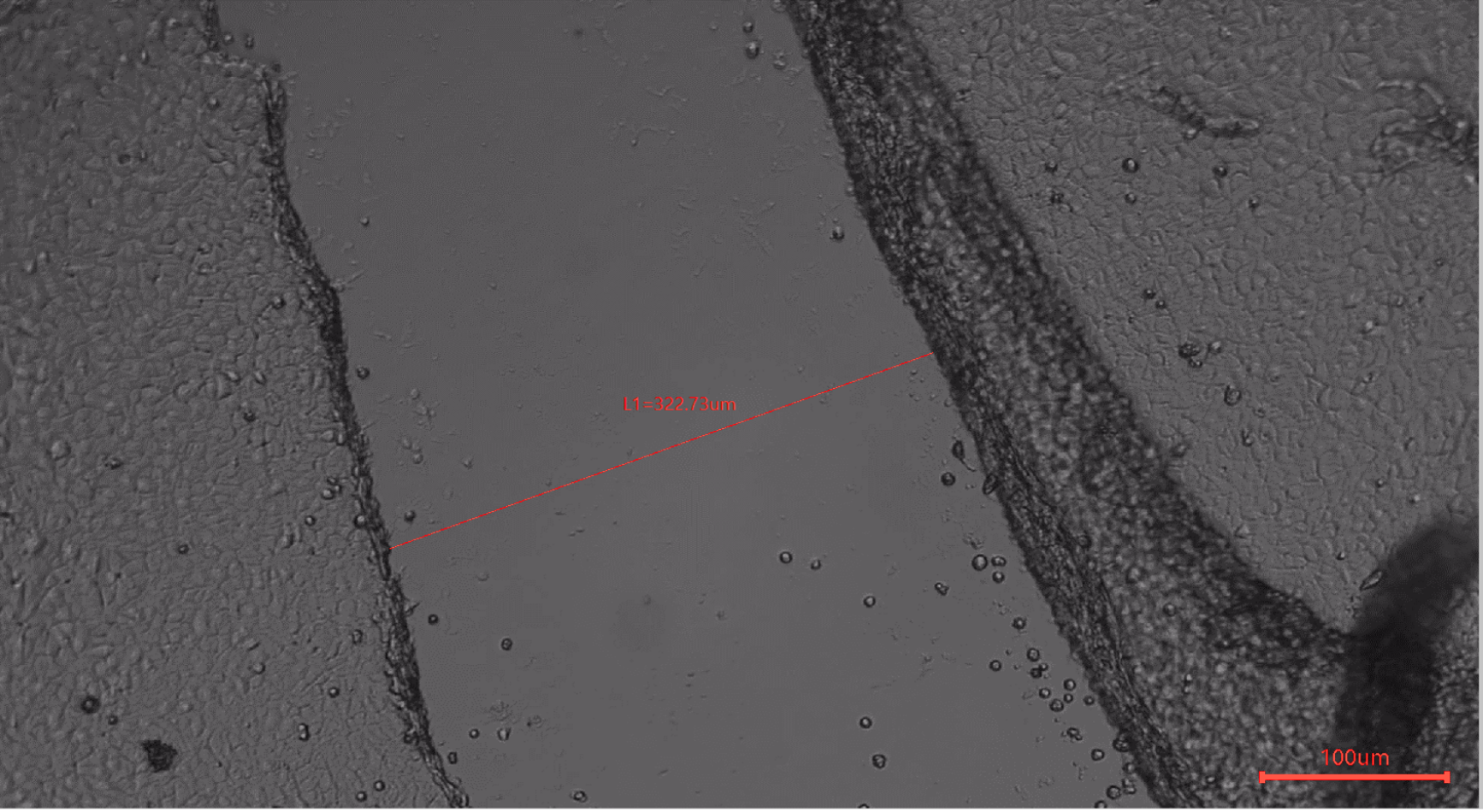

Supplement: Supplementary file 1 [file ijms-26-09485-s001.zip › Figure S3 Original image of the re-epithelialisation process representing E-T combi at time 0h.png]

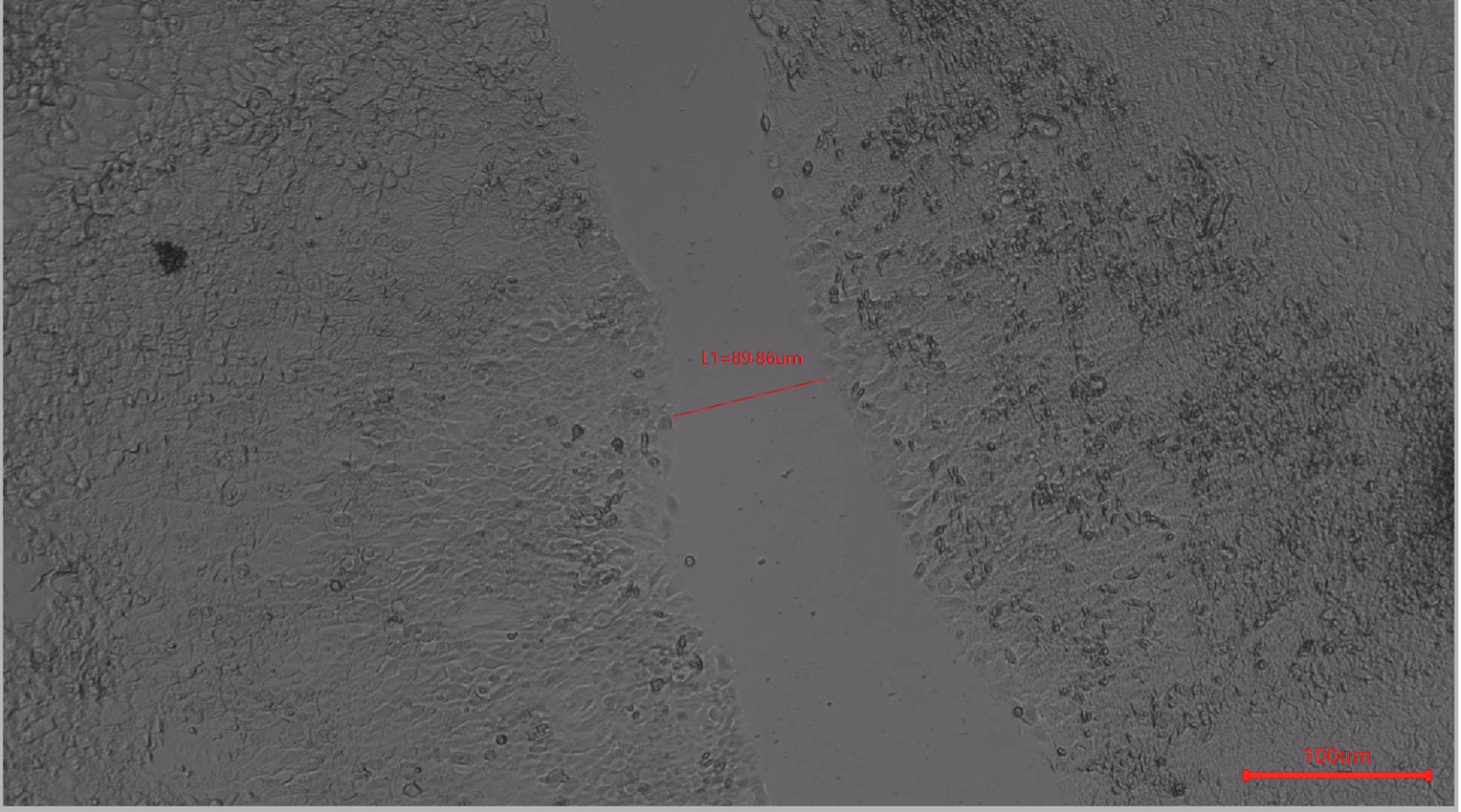

Supplement: Supplementary file 1 [file ijms-26-09485-s001.zip › Figure S4 Original image of the re-epithelialisation process representing E-T combi at time 24h.png]

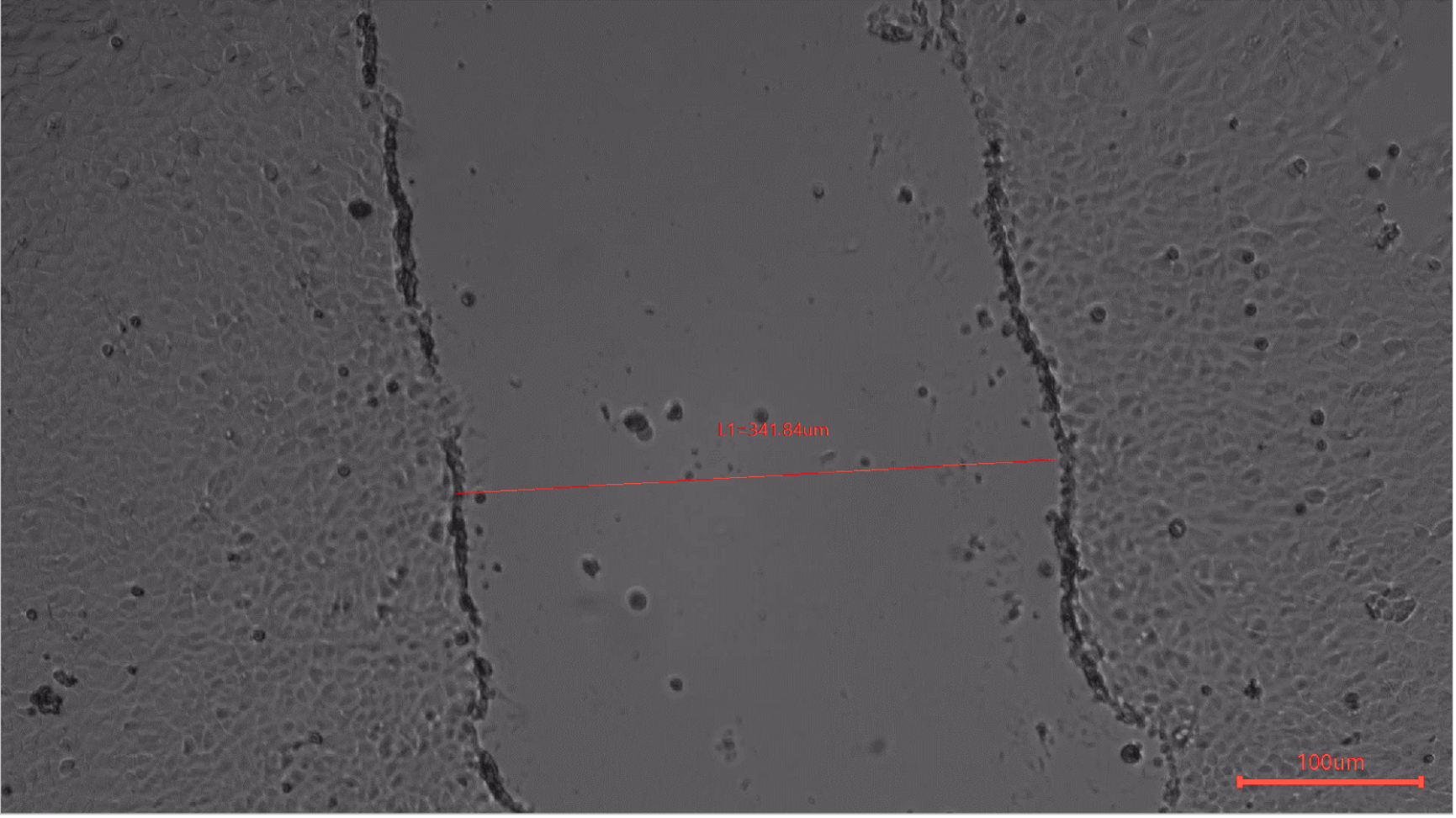

Supplement: Supplementary file 1 [file ijms-26-09485-s001.zip › Figure S5 Original image of the re-epithelialisation process representing positive control at time 0h.png]

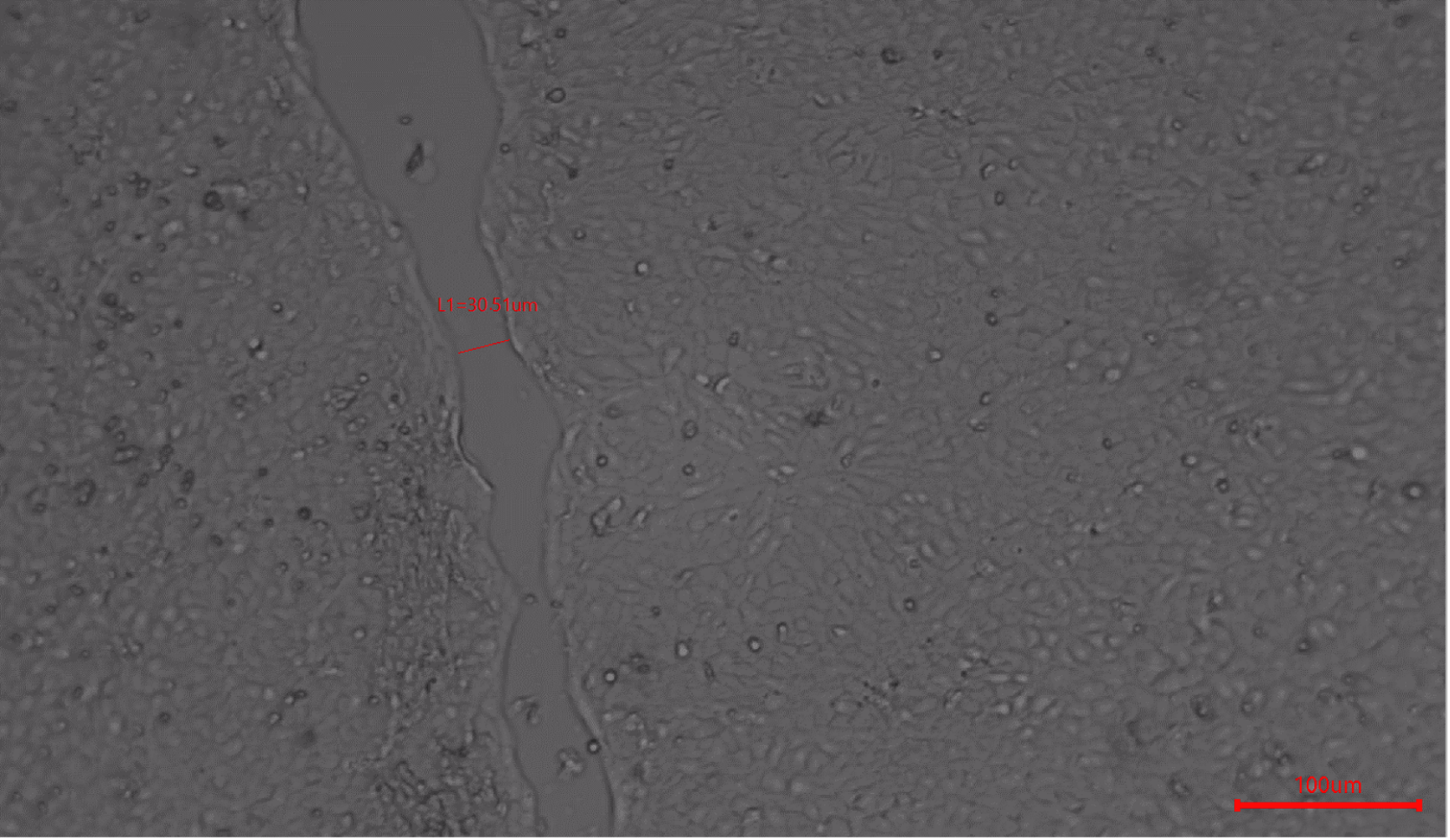

Supplement: Supplementary file 1 [file ijms-26-09485-s001.zip › Figure S6 Original image of the re-epithelialisation process representing positive control at time 24h.png]

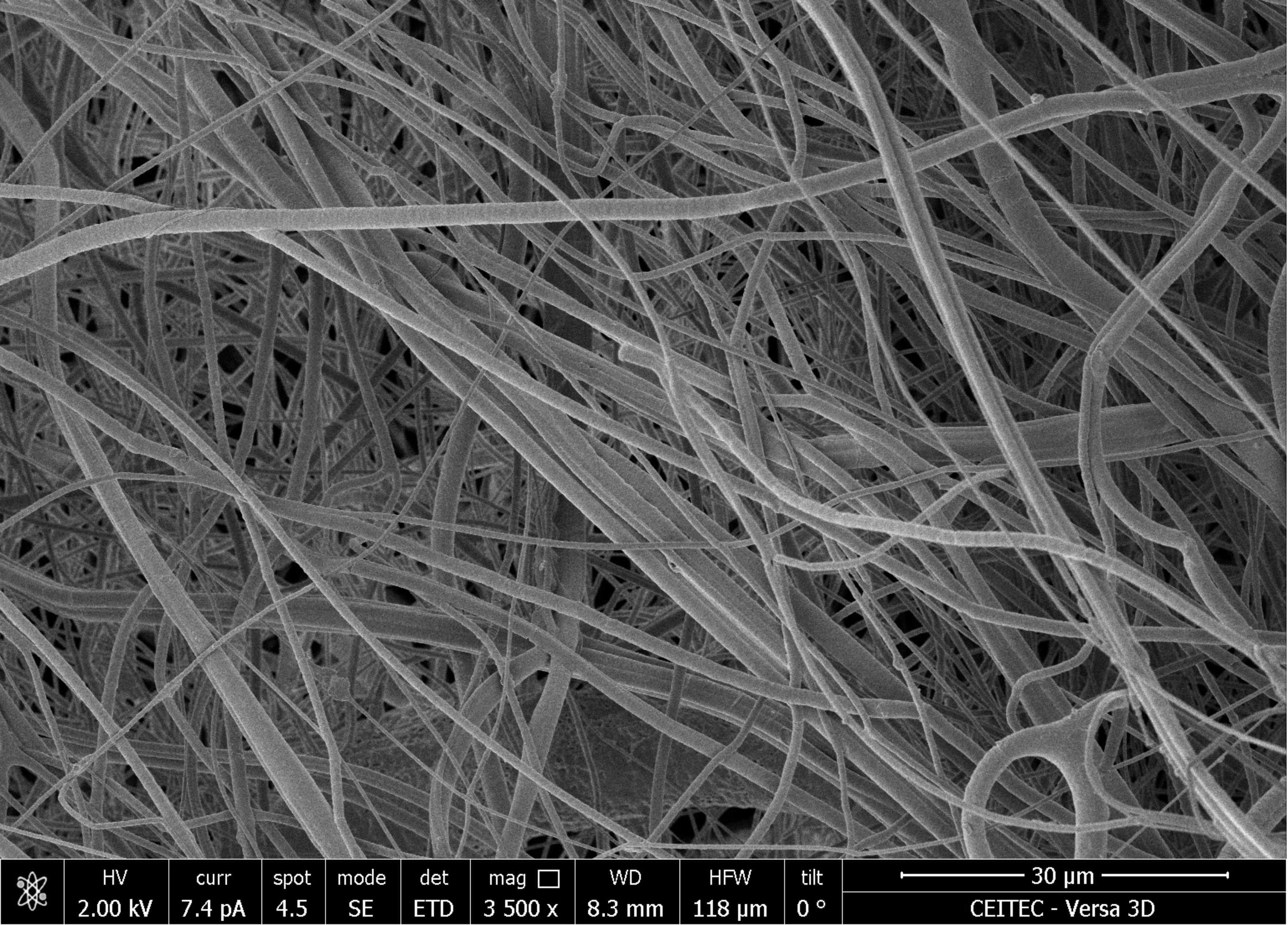

Supplement: Supplementary file 1 [file ijms-26-09485-s001.zip › Figure S7 Original SEM image of the morphology of the forcespun PHB fibres (A1) represented in Figure 2. A1.png]

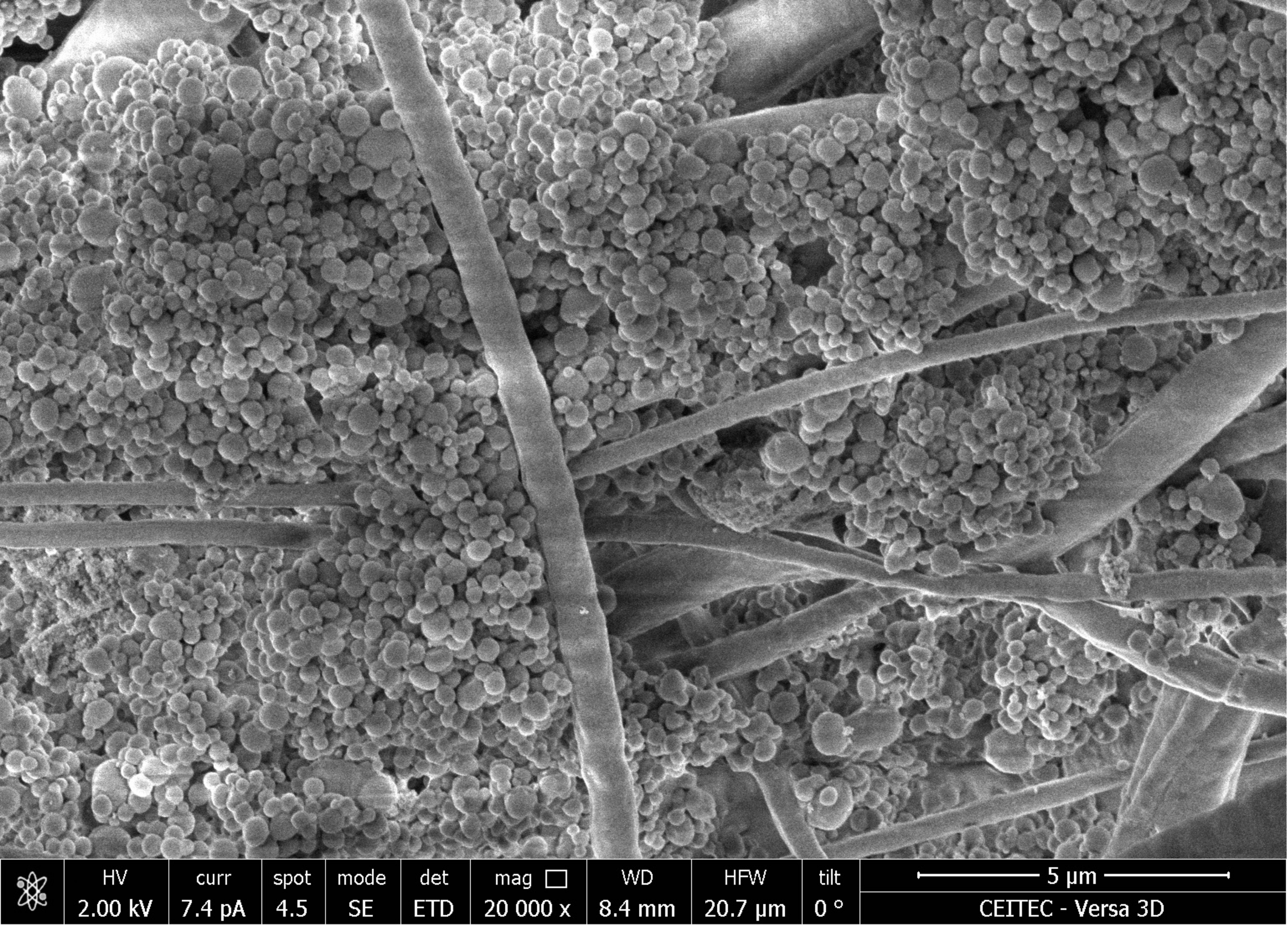

Supplement: Supplementary file 1 [file ijms-26-09485-s001.zip › Figure S8 Original SEM image of the morphology of the combined fibre-liposome structure (B1) represented in Figure 2. B1.png]

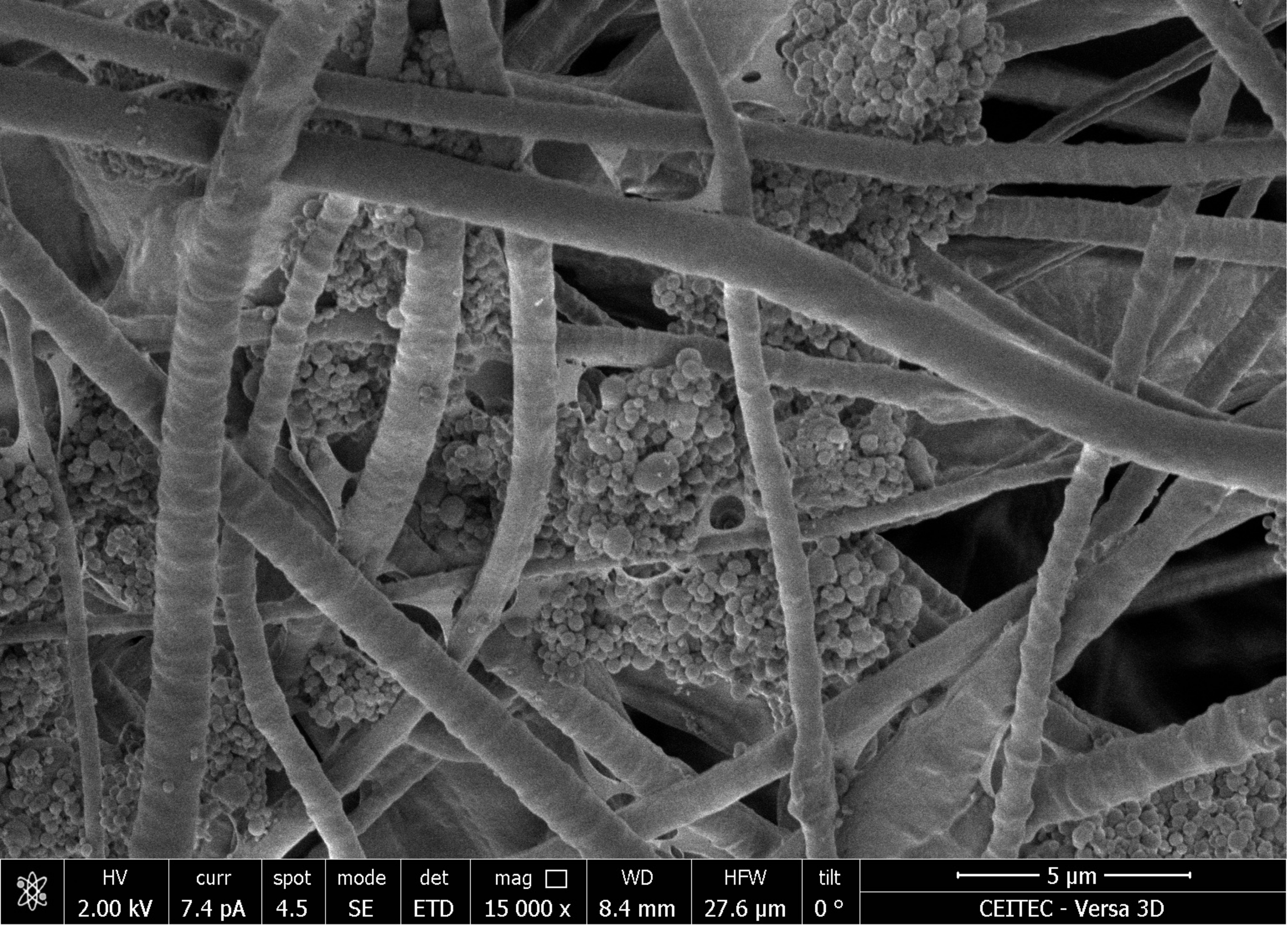

Supplement: Supplementary file 1 [file ijms-26-09485-s001.zip › Figure S9 Original SEM image of the morphology of the combined fibre-liposome structure (B2) represented in Figure 2. B2.png]
